# Structural characterization of two solvates of a luminescent copper(II) bis­(pyridine)-substituted benzimidazole complex

**DOI:** 10.1107/S2056989017014232

**Published:** 2017-10-06

**Authors:** David K. Geiger, Matthew R. DeStefano, Robert A. Lewis

**Affiliations:** a Department of Chemistry, SUNY-College at Geneseo, Geneseo, NY 14454, USA

**Keywords:** crystal structure, copper(II), benz­imidazole, hydrogen bonding, π–π inter­actions, C—H⋯π inter­actions

## Abstract

Copper(II) complexes of benzimidazole are known to exhibit biological activity that makes them of inter­est for chemotherapeutic and other pharmaceutical uses. The complex bis­(acetato-κ*O*){5,6-dimethyl-2-(pyridin-2-yl)-1-[(pyridin-2-yl)meth­yl]-1*H*-benzimidazole-κ^2^ *N* ^2^,*N* ^3^}copper(II), has been prepared. The absorption spectrum has features attributed to intra­ligand and ligand-field transitions and the complex exhibits ligand-centered room-temperature luminescence in solution. The aceto­nitrile monosolvate, [Cu(C_2_H_3_O_2_)_2_(C_20_H_18_N_4_)]·C_2_H_3_N (**1**), and the ethanol hemisolvate, [Cu(C_2_H_3_O_2_)_2_(C_20_H_18_N_4_)]·0.5C_2_H_6_O (**2**), have been structurally characterized. Compound **2** has two copper(II) complexes in the asymmetric unit. In both **1** and **2**, distorted square-planar N_2_O_2_ coordination geometries are observed and the Cu—N(Im) bond distance is slightly shorter than the Cu—N(py) bond distance. Inter­molecular π–π inter­actions are found in **1** and **2**. A weak C—H⋯π inter­action is observed in **1**.

## Abstract 2

The aceto­nitrile solvate and the ethanol hemisolvate of bis­(acetato-κ*O*){5,6-dimethyl-2-(pyridin-2-yl)-1-[(pyridin-2-yl)meth­yl]-1*H*-benzimidazole-κ^2^ *N* ^2^,*N* ^3^}copper(II) have been structurally characterized. Both compounds exhibit π–π inter­actions.

## Chemical context   

Copper(II) complexes containing benzimidazole ligands exhibit anti­cancer properties involving reactive oxygen species and DNA inter­actions (Prosser *et al.*, 2017 ▸; Lewis *et al.*, 2016 ▸; Mal *et al.*, 2014 ▸). Similar complexes show anti­bacterial activity (Chen *et al.*, 2012 ▸). The biological activity suggests that Cu^II^–benzimidazole complexes have potential as chemo­thera­peutic and other pharmaceutical uses.

In addition to biological applications, Cu^II^ complexes containing benzimidazole have been explored as catalysts. For example, one complex behaves as a ring-opening polymerization catalyst (Zaca *et al.*, 2016 ▸). Others have been used as building blocks for the construction of metal–organic frameworks and coordination polymers (Li *et al.*, 2011 ▸; Machura *et al.*, 2010 ▸).

We recently reported the structures of two zinc(II) complexes of 5,6-dimethyl-2-(pyridin-2-yl)-1-[(pyridin-2-yl)meth­yl]-1*H*-benzimidazole, Me_2_BzImpy_2_, that exhibit blue luminescence (DeStefano & Geiger, 2016 ▸) and a luminescent platinum(II) complex that exhibits an inter­molecular anagostic inter­action (DeStefano & Geiger, 2017 ▸). In this report, we expand the series to Cu^II^(Me_2_BzImpy_2_)(OAc)_2_, which is luminescent in solution. Two forms of the compound have been structurally characterized: **1** is an aceto­nitrile solvate and **2** is an ethanol hemisolvate.
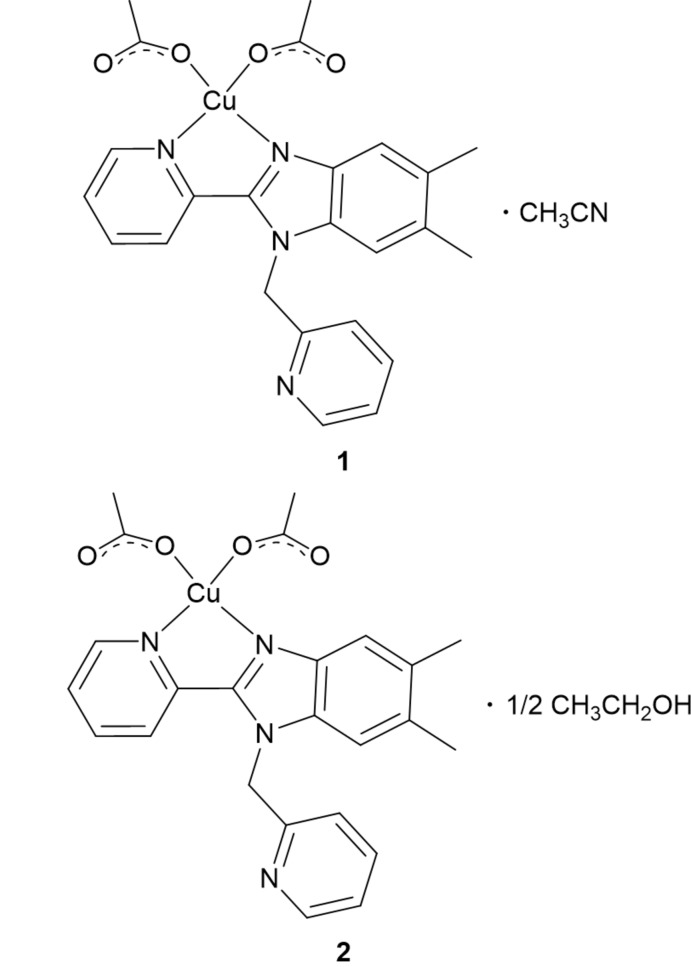


## Spectroscopy   

**Figure 1:**
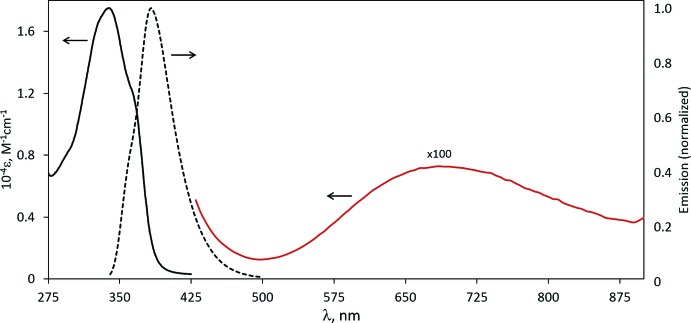
Absorption (solid black) and normalized emission (dash black, λ_exc_ = 320 nm) spectra for 5.25E-5 *M* Cu(Me_2_BzImpy_2_)(OAc)_2_. Absorption (solid red) spectrum for 5.25E-3 *M* Cu(Me_2_BzImpy_2_)(OAc)_2_ in ethanol.

The absorption and emission spectra of Cu(Me_2_BzImpy_2_)(OAc)_2_ are shown in Fig. 1 ▸. In the UV region, the absorption spectrum is similar to that of the free ligand, Me_2_BzImpy_2_, (Geiger & DeStefano, 2016 ▸) but red-shifted (λ_max_ = 340 nm, 3.65 eV; ∊ = 17,500 *M* ^−1^cm^−1^), as was observed for the zinc(II) (DeStefano & Geiger, 2016 ▸) and platinum(II) (DeStefano & Geiger, 2017 ▸) complexes of Me_2_BzImpy_2_. In the previously reported complexes, the bands were assigned as ligand-centered π*←π in nature based on the results of mol­ecular orbital calculations. In addition to the features in the UV region of the spectrum, a ligand-field band is observed in the visible region (λ_max_ = 695 nm, 1.78 eV; ∊ = 77 *M* ^−1^cm^−1^). The emission spectrum obtained using an excitation wavelength of 320 nm exhibits a band (λ_max_ = 383 nm, 3.24 eV) similar to those of the Zn^II^ and Pt^II^ complexes where there is evidence of involvement of the di­imine in the emissive state (DeStefano & Geiger, 2017 ▸; Hissler *et al.*, 2000 ▸).

Luminescent Cu^II^ 1,10-phenanthroline complexes have been reported (Melnic *et al.*, 2014 ▸; Mistri, García-Granda *et al.*, 2013 ▸; Mistri, Zangrando *et al.*, 2013 ▸). In these complexes, ligand-field transitions appear in the near infrared, which do not exhibit emission bands due to ultrafast non-radiative processes (Melnic *et al.*, 2014 ▸). Bis(1,2-benzenedi­amine-κ^2^ *N,N′*)copper(II) nitrate (Supriya & Das, 2003 ▸) and a series of copper(II) complexes with tridentate phenol-substituted picolinylidenes (Das & Pal, 2010 ▸) are other examples of luminescent Cu^II^ complexes. However, to our knowledge, Cu(Me_2_BzImpy_2_)(OAc)_2_ is the first luminescent Cu^II^–benz­im­idazole complex reported.

## Structural commentary   

**Figure 2:**
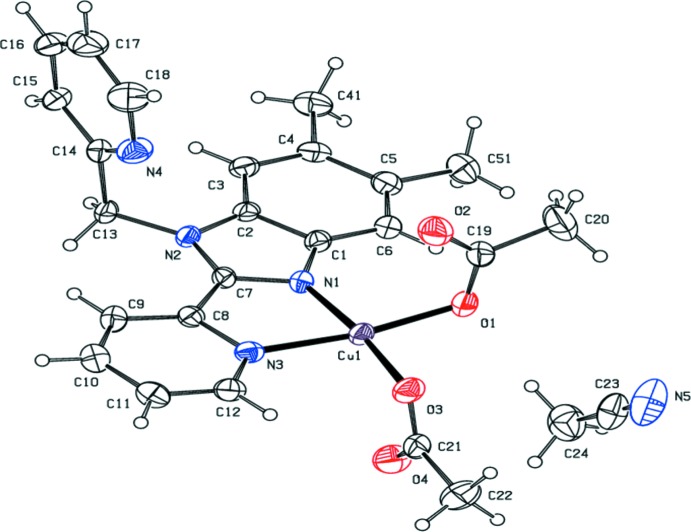
View of **1** showing the atom-labeling scheme. Anisotropic displacement parameters of non-H atoms are drawn at the 30% probability level.

The two copper complexes explored in this study differ in the co-crystallized solvent: **1** contains one aceto­nitrile mol­ecule per copper complex, whereas **2** is an ethanol solvate with two symmetry-independent mol­ecules of the copper complex per mol­ecule of ethanol. The two independent mol­ecules will be referred to as **2** ***a*** and **2** ***b***. Representations of the asymmetric units of **1** and **2** along with the respective atom-labeling schemes are found in Figs. 2 ▸ and 3 ▸. The ethanol mol­ecule in **2** is threefold disordered (see *Refinement* section for details).

In both **1** and **2**, the coordination geometries of the copper ions are best described as distorted square planar with monodentate coordination of two acetate ligands in addition to the Me_2_BzImpy_2_ ligand (see Figs. 2 ▸ and 3 ▸, and Tables 1 ▸ and 2 ▸). In **1**, the uncoordinated oxygen atoms are 2.651 (3) and 2.676 (4) Å from the Cu^II^ atom. In **2** ***a***, the corresponding distances are 2.471 (2) and 2.698 (3) Å; in **2** ***b***, the distances are 2.546 (3) and 2.554 (3) Å. The oxygen atoms of the N_2_O_2_ coordination sphere have a twist angle from the nitro­gen atoms of 6.7 (2)° for **1**. These values are 17.2 (2) and 7.9 (2)° for **2** ***a*** and **2** ***b***, respectively. In **1**, **2** ***a*** and **2** ***b***, the two acetate ligands adopt *anti* conformations.

The Cu—N(pyridine) bond distances found in **1** and **2** are slightly longer than the Cu—N(imidazole) bond distances (Tables 1 ▸ and 2 ▸). The bond distances compare favorably with those found in other Cu^II^ 2-pyridin-2-yl-1*H*-benzimidazole complexes. For the four square-planar complexes in the comparison pool (Prosser *et al.*, 2017 ▸; Lewis *et al.*, 2016 ▸; Li *et al.*, 2011 ▸), the Cu—N(pyridine) bond distances are 2.04 (2) Å [range = 2.0047 (2)–2.059 (4) Å] and the Cu—N(imidazole) bond distances are 1.99 (2) Å [range = 1.9645 (2)–2.002 (2) Å].

In **1** and **2**, the coordinated pyridine and benzimidazole ring systems are approximately coplanar. The torsion angles are reported in Tables 1 ▸ and 2 ▸. In **1** the angle between the mean planes of the benzimidazole ring system and the coordinated pyridine is 0.89 (19)° and in **2** ***a*** and **2** ***b*** the corresponding angles are 3.5 (2) and 4.91 (16)°, respectively.

## Supra­molecular features   

**Figure 4:**
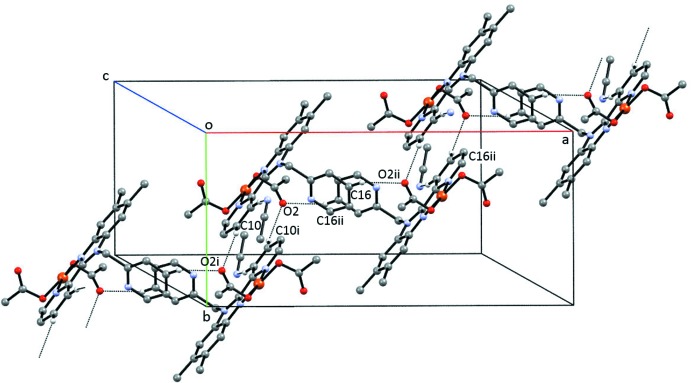
Partial packing diagram of **1** showing the chains formed by C—H⋯O hydrogen bonding. Only the H atoms involved in the inter­actions are shown. [Symmetry codes: (i) −*x* + 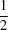, −*y* + 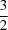, −*z* + 1; (iv) *x* + 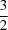, *y* + 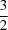, *z* + 1.]

There are several types of hydrogen-bonding inter­actions present in **1**, as seen in Table 3 ▸. The aceto­nitrile solvate participates as acceptor in C—H⋯N hydrogen bonds in which an aromatic hydrogen atom (H9) is donor. Additionally, C—H⋯O hydrogen bonds involving the uncoordinated acetate oxygen atom O2 as acceptor and the aromatic carbon atoms C10 and C16 as donors result in chains that run parallel to [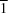 10] (Fig. 4 ▸). In **2**, the most significant hydrogen-bonding inter­actions (Table 4 ▸) involve the disordered ethanol solvate mol­ecule, which participates as donor in O—H⋯O hydrogen bonding with the uncoordinated acetate oxygen atoms O2 and O6 as acceptors.

**Figure 5:**
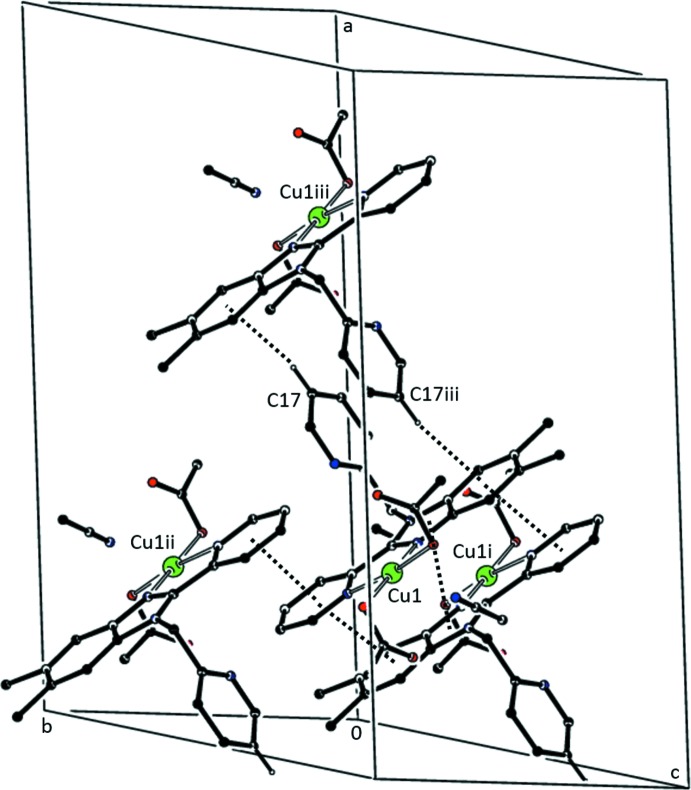
Partial packing diagram of **1** showing the C—H⋯π and the primary π–π inter­actions. Only the H atom involved in the C—H⋯π inter­action is shown. [Symmetry identifiers: (i) −*x* + 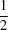, −*y* + 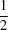, −*z* + 1; (ii) −*x* + 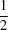, −*y* + 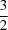, −*z* + 1; (iii) −*x* + 1, −*y* + 1, −*z* + 1.]

In addition to weak C—H⋯O and C—H⋯N hydrogen bonds (Table 3 ▸), the extended structure of **1** exhibits inter­molecular C—H⋯π and π–π inter­actions (see Tables 3 ▸ and 5 ▸). Fig. 5 ▸ shows a partial packing diagram emaphasizing these inter­actions. The closest π–π inter­action exists between the coordinated pyridine rings of mol­ecules related by a crystallographically imposed inversion center. Weaker π–π inter­actions exist between imidazole rings and between coordin­ated pyridine rings and benzene rings on inversion-related mol­ecules. The C—H⋯π inter­action is between inversion-related mol­ecules and involves the benzene ring and a hydrogen atom of the 1-(pyridin-2-yl)methyl substituent.

**Figure 6:**
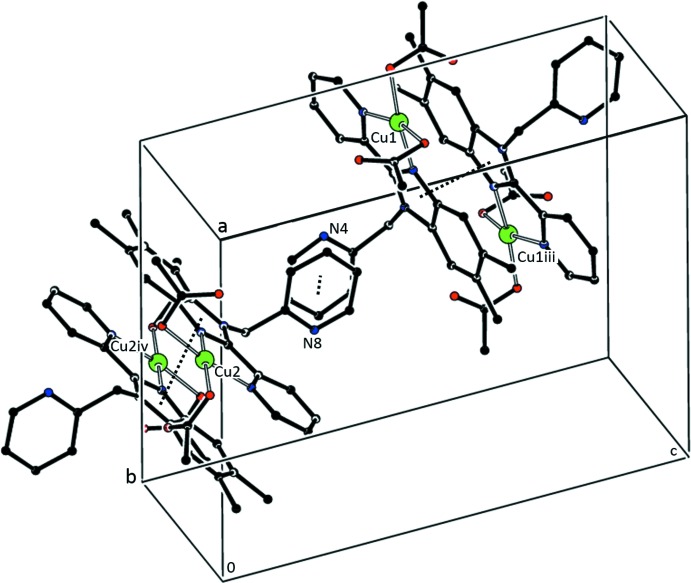
Partial packing diagram of **2** emphasizing the primary π–π inter­actions. H atoms and the ethanol solvate mol­ecule are not shown. [Symmetry identifiers: (iii) −*x* + 2, −*y*, −*z* + 1; (iv) −*x* + 1, −*y* + 1, −*z*.]

No significant C—H⋯π inter­actions are observed in **2**; however, a number of close π–π inter­actions exist (see Table 5 ▸ and Fig. 6 ▸). A π–π inter­action between **2** ***a*** and **2** ***b*** involves the 1-(pyridin-2-yl)methyl substituent of each mol­ecule. In both **2** ***a*** and **2** ***b***, the closest π–π inter­action is between imidazole rings related by the crystallographically imposed inversion center. The *Cg*(Im)⋯*Cg*(Im) separation in **2a** is shorter than that observed in **2b**.

π stacking is prevalent in Cu^II^ 1,10-phenanthroline complexes (Melnic *et al.*, 2014 ▸) and it has been suggested as a necessary structural feature for the DNA-cleavage activity exhibited by these and similar complexes (McCann *et al.*, 2013 ▸). π stacking has also been implicated in the fluorescence quenching of amyloid-*β* peptide, which could be of relevance to possible therapeutic applications of Cu^II^ chelators in the treatment of Alzheimer’s disease (Melnic *et al.*, 2014 ▸).

## Database survey   

A search of the Cambridge Structural Database (*WebCSD*; Groom *et al.*, 2016 ▸) for 2-(pyridin-2-yl)-1*H*-benzimidazole ligands coordinated to Cu^II^ yielded 14 different compounds. The most similar to **1** and **2** are the four which adopt square-planar coordination geometries (EQOGAT: Li *et al.*, 2011 ▸; MALLAP: Lewis *et al.*, 2016 ▸; CANMIQ and CANMUC: Prosser *et al.*, 2017 ▸). Two complexes exhibit octa­hedral coordination geometries (MALLUJ: Lewis *et al.*, 2016 ▸; TUBXUK: Altaf & Stoeckli-Evans, 2009 ▸), five have square-pyramidal geometries (RAXQUE: Chen *et al.*, 2012 ▸; BUYCUU: Machura *et al.*, 2010 ▸; ZOTCUI: Mal *et al.*, 2014 ▸; GUXBOR: Zhang & Yang, 2010 ▸; COXSOY01: Altaf & Stoeckli-Evans, 2009 ▸), and three have trigonal–bipyramidal geometries (CANMEM and CANMOW: Prosser *et al.*, 2017 ▸; OVAXEQ: Zaca *et al.*, 2016 ▸). Excluding those complexes exhibiting Jahn–Teller distorted geometries, the average Cu—N(pyridine) and Cu—N(imidazole) bond distances found are 2.04 (2) and 2.00 (4) Å, respectively.

## Synthesis and crystallization   

5,6-Dimethyl-2-(pyridin-2-yl)-1-[(pyridin-2-yl)meth­yl]-1*H*-benzimidazole, Me_2_BzImpy_2_, was prepared as previously described (Geiger & DeStefano, 2014 ▸). Solvents were of commercial analytical grade and used without further purification. Spectroscopic measurements were performed at ambient temperature. Absorption spectra were recorded on a Varian Cary 50 Bio UV–Visible spectrophotometer. Excitation and emission spectra were recorded on a Photon Technology Inter­national Inc. QM-40 spectrofluorimeter using an excitation wavelength of 320 nm.

Bis(acetato-κ*O*){5,6-dimethyl-2-(pyridin-2-yl)-1-[pyridin-2-yl)meth­yl]-1*H*-benzimidazole-κ^2^ *N* ^2^,*N* ^3^}copper(II), Cu(Me_2_BzImpy_2_)(OAc)_2_, was prepared by refluxing 200 mg (0.63 mmol) Me_2_BzImpy_2_ and 130 mg (0.65 mmol) copper acetate monohydrate in 15 mL ethanol for 10 min. The ethanol was reduced in volume until crystallization commenced. After chilling in an ice bath, the blue crystalline product was separated by filtration. The yield was 0.24 g (0.52 mmol, 83% yield). Single crystals of **1** and **2** were obtained by slow evaporation of aceto­nitrile and ethanol solutions of Cu(Me_2_BzImpy_2_)(OAc)_2_, respectively.

## Refinement   

Crystal data, data collection and structure refinement details are summarized in Table 6 ▸. Early in the refinement of **2**, the ethanol hemisolvate mol­ecule was found to be disordered. The disorder was modeled using three contributors. Successful refinement required the use of O—H, C—C and C—O distance restraints of 0.84, 1.53 and 1.43 Å, respectively, and restraints on the *U^ij^* components of the anisotropically refined atoms in the disordered ethanol mol­ecule. The disorder model refined to occupancies of 0.411 (3):0.362 (3):0.227 (3). All H atoms were located in difference-Fourier maps for **1** and **2**, except those associated with the disordered ethanol mol­ecule. H atoms bonded to C atoms were refined using a riding model, with C—H = 0.95 Å and *U*_iso_(H) = 1.2*U*_eq_(C) for the aromatic positions; C—H = 0.99 Å and *U*_iso_(H) = 1.2*U*_eq_(C) for the methyl­ene groups; and C—H = 0.98 Å and *U*_iso_(H) = 1.5*U*_eq_(C) for the methyl groups. The hy­droxy H atoms in the disordered ethanol contributors were refined using a rotating-group model with C—O—H tetra­hedral, distance restraints to acceptor atoms (O6 and symmetry-generated O2) and with *U*_iso_(H) = 1.5*U*_eq_(O).

## Supplementary Material

Crystal structure: contains datablock(s) 1, 2. DOI: 10.1107/S2056989017014232/zl2717sup1.cif

Structure factors: contains datablock(s) 1. DOI: 10.1107/S2056989017014232/zl27171sup2.hkl

Supporting information file. DOI: 10.1107/S2056989017014232/zl27171sup4.mol

Structure factors: contains datablock(s) 2. DOI: 10.1107/S2056989017014232/zl27172sup3.hkl

Supporting information file. DOI: 10.1107/S2056989017014232/zl27172sup5.mol

CCDC references: 1577748, 1577747

Additional supporting information:  crystallographic information; 3D view; checkCIF report

## Figures and Tables

**Figure 3:**
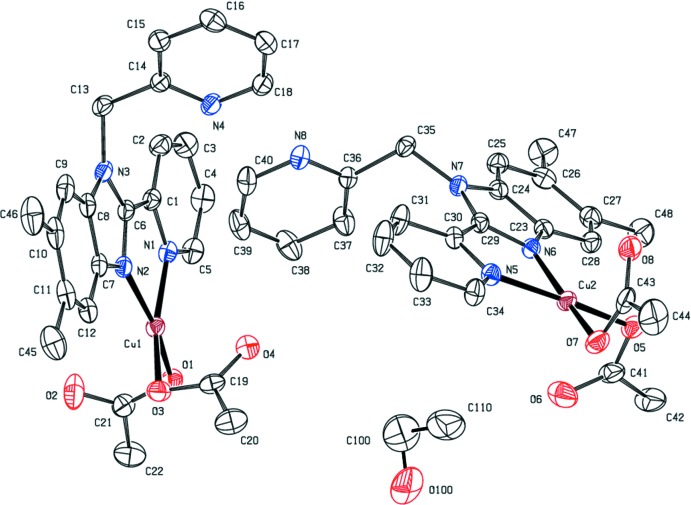
View of **2** showing the atom-labeling scheme. Anisotropic displacement parameters of non-H atoms are drawn at the 30% probability level. Hydrogen atoms are not shown. Only the major contributor to the disordered ethanol mol­ecule is shown.

**Table 1:** Selected geometric parameters (Å, °) for **1** [Chem scheme1]

Cu1—N1	1.974 (3)	Cu1—O1	1.948 (3)
Cu1—N3	2.034 (3)	Cu1—O3	1.935 (2)
			
N1—Cu1—N3	80.41 (11)	O3—Cu1—N3	93.35 (12)
O1—Cu1—N3	171.88 (11)	O3—Cu1—N1	173.39 (12)
O1—Cu1—N1	94.59 (12)	O3—Cu1—O1	91.85 (12)
			
N1—C7—C8—N3	0.0 (5)		

**Table 2:** Selected geometric parameters (Å, °) for **2** [Chem scheme1]

Cu1—N1	2.024 (3)	Cu2—N5	2.029 (2)
Cu1—N2	1.962 (2)	Cu2—N6	1.987 (3)
Cu1—O1	1.931 (2)	Cu2—O5	1.961 (2)
Cu1—O3	1.974 (2)	Cu2—O7	1.955 (2)
			
O1—Cu1—N2	166.81 (11)	O7—Cu2—O5	94.38 (10)
O1—Cu1—O3	94.41 (9)	O7—Cu2—N6	170.19 (10)
N2—Cu1—O3	94.24 (10)	O5—Cu2—N6	93.55 (10)
O1—Cu1—N1	93.50 (10)	O7—Cu2—N5	92.34 (10)
N2—Cu1—N1	80.25 (10)	O5—Cu2—N5	172.00 (11)
O3—Cu1—N1	165.68 (10)	N6—Cu2—N5	80.24 (10)
			
N1—C1—C6—N2	1.2 (4)		

**Table 3:** Hydrogen-bond geometry (Å, °) for **1** [Chem scheme1] *Cg*(Bz) is the centroid of the benzene ring.

*D*—H⋯*A*	*D*—H	H⋯*A*	*D*⋯*A*	*D*—H⋯*A*
C12—H12⋯O3	0.95	2.51	3.043 (5)	115
C10—H10⋯O2^i^	0.95	2.42	3.140 (5)	132
C6—H6⋯O1	0.95	2.54	3.191 (5)	126
C24—H24*B*⋯O1	0.98	2.59	3.334 (6)	133
C9—H9⋯N5^ii^	0.95	2.50	3.349 (6)	149
C13—H13*B*⋯O4^iii^	0.99	2.41	3.391 (5)	169
C16—H16⋯O2^iv^	0.95	2.57	3.449 (6)	154
C17—H17⋯*Cg*(Bz)^v^	0.95	2.87	3.783 (6)	162

Symmetry codes: (i) 
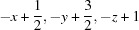
; (ii) 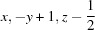; (iii) 
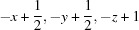
; (iv) 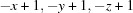; (v) 
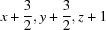
.

**Table 4:** Hydrogen-bond geometry (Å, °) for **2** [Chem scheme1]

*D*—H⋯*A*	*D*—H	H⋯*A*	*D*⋯*A*	*D*—H⋯*A*
O100—H100⋯O2^i^	0.84	2.25	3.010 (8)	152
O200—H200⋯O6	0.84	2.31	3.068 (14)	149
O300—H300⋯O6	0.84	2.41	3.13 (2)	144
C5—H5⋯N8^ii^	0.95	2.52	3.279 (4)	137
C13—H13*A*⋯O2^iii^	0.99	2.53	3.497 (4)	167
C17—H17⋯O8^iv^	0.95	2.63	3.334 (4)	132
C20—H20*B*⋯O3^i^	0.98	2.61	3.576 (5)	169
C25—H25⋯O8^iv^	0.95	2.45	3.321 (4)	152
C33—H33⋯O100^v^	0.95	2.51	3.202 (8)	130
C35—H35*A*⋯O8^iv^	0.99	2.37	3.353 (4)	170

Symmetry codes: (i) 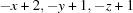; (ii) 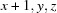; (iii) 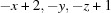; (iv) 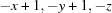; (v) 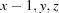.

**Table 5:** Significant π–π inter­actions (Å) in **1** and **2** *Cg*(*n*) refers to the centroids of the imidazole (*n* = Im), benzene (*n* = Bz), pyridine (*n* = py), and pyridyl­methyl (*n* = Pym) rings.

**1**		**2**	
Cg(Im)⋯Cg(Im)^i^	3.502 (2)	Cg(Pym**2a**)⋯Cg(Pym**2b**)	3.955 (2)
Cg(py)⋯Cg(py)^ii^	3.415 (2)	Cg(Im**2a**)⋯Cg(Im**2a**)^iii^	3.3405 (18)
Cg(py)⋯Cg(Bz)^ii^	3.603 (2)	Cg(Im**2b**)⋯Cg(Im**2b**)^iv^	3.5618 (19)

Symmetry codes: (i) −*x* + 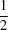, −*y* + 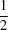, 1 − *z*; (ii) −*x* + 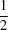, −*y* + 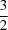, −*z* + 1; (iii) −*x* + 2, −*y*, −*z* + 1; (iv) −*x* + 1, −*y* + 1, −*z*.

**Table 6:** Experimental details

	**1**	**2**
Crystal data		
Chemical formula	[Cu(C_2_H_3_O_2_)_2_(C_20_H_18_N_4_)]·C_2_H_3_N	[Cu(C_2_H_3_O_2_)_2_(C_20_H_18_N_4_)]·0.5C_2_H_6_O
*M*_r_	537.06	1038.09
Crystal system, space group	Monoclinic, *C*2/*c*	Triclinic, *P* 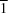
Temperature (K)	200	200
*a*, *b*, *c* (Å)	21.292 (3), 10.1837 (12), 24.867 (4)	11.2595 (11), 14.0130 (15), 16.5004 (18)
α, β, γ (°)	90, 102.668 (5), 90	81.873 (4), 77.126 (4), 80.348 (4)
*V* (Å^3^)	5260.7 (12)	2487.4 (5)
*Z*	8	2
Radiation type	Mo *K*α	Mo *K*α
μ (mm^−1^)	0.87	0.92
Crystal size (mm)	0.60 × 0.15 × 0.15	0.60 × 0.30 × 0.20

Data collection		
Diffractometer	Bruker SMART X2S benchtop	Bruker SMART X2S benchtop
Absorption correction	Multi-scan (*SADABS*; Bruker, 2013 ▸)	Multi-scan (*SADABS*; Bruker, 2013 ▸)
*T*_min_, *T*_max_	0.855, 0.878	0.726, 0.832
No. of measured, independent and observed [*I* > 2σ(*I*)] reflections	17879, 4616, 3415	22519, 9149, 6690
*R*_int_	0.093	0.042
(sin θ/λ)_max_ (Å^−1^)	0.595	0.610

Refinement		
*R*[*F* ^2^ > 2σ(*F* ^2^)], *wR*(*F* ^2^), *S*	0.059, 0.178, 1.02	0.045, 0.121, 1.04
No. of reflections	4616	9149
No. of parameters	330	690
No. of restraints	0	160
H-atom treatment	H-atom parameters constrained	H-atom parameters constrained
Δρ_max_, Δρ_min_ (e Å^−3^)	0.84, −1.27	0.77, −0.42

Computer programs: *APEX2* and *SAINT* (Bruker, 2013 ▸), *SHELXS97* (Sheldrick, 2008 ▸), *SHELXL2014* (Sheldrick, 2015 ▸), *PLATON* (Spek, 2009 ▸), *Mercury* (Macrae *et al.*, 2006 ▸) and *publCIF* (Westrip, 2010 ▸).

## supplementary crystallographic information

### Bis(acetato-κO){5,6-dimethyl-2-(pyridin-2-yl)-1-[(pyridin-2-yl)methyl]-1H-benzimidazole-κ2N2,N3}copper(II) acetonitrile monosolvate (1) Crystal data

**Table:**

[Cu(C_2_H_3_O_2_)_2_(C_20_H_18_N_4_)]·C_2_H_3_N	*F*(000) = 2232
*M**_r_* = 537.06	*D*_x_ = 1.356 Mg m^−^^3^
Monoclinic, *C*2/*c*	Mo *K*α radiation, λ = 0.71073 Å
*a* = 21.292 (3) Å	Cell parameters from 6746 reflections
*b* = 10.1837 (12) Å	θ = 2.3–25.6°
*c* = 24.867 (4) Å	µ = 0.87 mm^−^^1^
β = 102.668 (5)°	*T* = 200 K
*V* = 5260.7 (12) Å^3^	Needle, blue-green
*Z* = 8	0.60 × 0.15 × 0.15 mm

### Bis(acetato-κO){5,6-dimethyl-2-(pyridin-2-yl)-1-[(pyridin-2-yl)methyl]-1H-benzimidazole-κ2N2,N3}copper(II) acetonitrile monosolvate (1) Data collection

**Table:**

Bruker SMART X2S benchtop diffractometer	3415 reflections with *I* > 2σ(*I*)
Radiation source: sealed microfocus tube	*R*_int_ = 0.093
ω scans	θ_max_ = 25.0°, θ_min_ = 2.2°
Absorption correction: multi-scan (SADABS; Bruker, 2013)	*h* = −25→25
*T*_min_ = 0.855, *T*_max_ = 0.878	*k* = −11→11
17879 measured reflections	*l* = −16→29
4616 independent reflections	

### Bis(acetato-κO){5,6-dimethyl-2-(pyridin-2-yl)-1-[(pyridin-2-yl)methyl]-1H-benzimidazole-κ2N2,N3}copper(II) acetonitrile monosolvate (1) Refinement

**Table:**

Refinement on *F*^2^	Primary atom site location: structure-invariant direct methods
Least-squares matrix: full	Secondary atom site location: difference Fourier map
*R*[*F*^2^ > 2σ(*F*^2^)] = 0.059	Hydrogen site location: inferred from neighbouring sites
*wR*(*F*^2^) = 0.178	H-atom parameters constrained
*S* = 1.02	*w* = 1/[σ^2^(*F*_o_^2^) + (0.1166*P*)^2^] where *P* = (*F*_o_^2^ + 2*F*_c_^2^)/3
4616 reflections	(Δ/σ)_max_ = 0.003
330 parameters	Δρ_max_ = 0.84 e Å^−^^3^
0 restraints	Δρ_min_ = −1.27 e Å^−^^3^

### Bis(acetato-κO){5,6-dimethyl-2-(pyridin-2-yl)-1-[(pyridin-2-yl)methyl]-1H-benzimidazole-κ2N2,N3}copper(II) acetonitrile monosolvate (1) Special details

**Table:**

Geometry. All esds (except the esd in the dihedral angle between two l.s. planes) are estimated using the full covariance matrix. The cell esds are taken into account individually in the estimation of esds in distances, angles and torsion angles; correlations between esds in cell parameters are only used when they are defined by crystal symmetry. An approximate (isotropic) treatment of cell esds is used for estimating esds involving l.s. planes.
Refinement. All H atoms were located in difference Fourier maps. H atoms bonded to C atoms were refined using a riding model, with C–H = 0.95 Å and Uiso(H) = 1.2Ueq(C) for the aromatic positions; C–H = 0.99 Å and Uiso(H) = 1.2Ueq(C) for the methylene group; and C–H = 0.98 Å and Uiso(H) = 1.5Ueq(C) for the methyl groups.

### Bis(acetato-κO){5,6-dimethyl-2-(pyridin-2-yl)-1-[(pyridin-2-yl)methyl]-1H-benzimidazole-κ2N2,N3}copper(II) acetonitrile monosolvate (1) Fractional atomic coordinates and isotropic or equivalent isotropic displacement parameters (Å^2^)

**Table:**

	*x*	*y*	*z*	*U*_iso_*/*U*_eq_	
Cu1	0.25894 (2)	0.51131 (4)	0.60501 (2)	0.0386 (2)	
O1	0.30689 (14)	0.4530 (3)	0.67694 (10)	0.0513 (7)	
O2	0.37267 (15)	0.6034 (3)	0.65669 (12)	0.0656 (8)	
O3	0.21236 (14)	0.6405 (3)	0.63799 (11)	0.0527 (7)	
O4	0.14226 (17)	0.4765 (3)	0.62642 (15)	0.0652 (9)	
N1	0.29915 (14)	0.3832 (3)	0.56335 (11)	0.0359 (7)	
N2	0.32326 (14)	0.3222 (3)	0.48426 (11)	0.0345 (7)	
N3	0.22120 (15)	0.5741 (3)	0.52699 (12)	0.0385 (7)	
N4	0.3977 (2)	0.5171 (3)	0.44721 (17)	0.0583 (10)	
N5	0.2435 (4)	0.5210 (6)	0.8052 (2)	0.112 (2)	
C1	0.34177 (17)	0.2802 (3)	0.57473 (13)	0.0368 (8)	
C2	0.35794 (17)	0.2411 (3)	0.52502 (13)	0.0358 (8)	
C3	0.40079 (18)	0.1394 (4)	0.52338 (15)	0.0431 (9)	
H3	0.4112	0.1143	0.4896	0.052*	
C4	0.42816 (18)	0.0748 (4)	0.57212 (16)	0.0455 (9)	
C5	0.41051 (19)	0.1134 (4)	0.62263 (16)	0.0487 (10)	
C6	0.36792 (18)	0.2129 (4)	0.62356 (14)	0.0435 (9)	
H6	0.3561	0.2363	0.6570	0.052*	
C7	0.28948 (17)	0.4081 (3)	0.50934 (13)	0.0342 (8)	
C8	0.24562 (19)	0.5156 (3)	0.48640 (15)	0.0357 (8)	
C9	0.2294 (2)	0.5596 (4)	0.43257 (14)	0.0442 (9)	
H9	0.2470	0.5186	0.4049	0.053*	
C10	0.1877 (2)	0.6624 (4)	0.41948 (16)	0.0509 (10)	
H10	0.1762	0.6934	0.3826	0.061*	
C11	0.1624 (2)	0.7209 (4)	0.45988 (16)	0.0507 (10)	
H11	0.1330	0.7920	0.4514	0.061*	
C12	0.18092 (19)	0.6736 (4)	0.51333 (16)	0.0465 (9)	
H12	0.1639	0.7145	0.5414	0.056*	
C13	0.33262 (18)	0.3199 (4)	0.42732 (13)	0.0389 (9)	
H13A	0.2938	0.3556	0.4021	0.047*	
H13B	0.3385	0.2281	0.4163	0.047*	
C14	0.39095 (18)	0.4007 (4)	0.42239 (14)	0.0414 (9)	
C15	0.43403 (19)	0.3528 (5)	0.39292 (16)	0.0554 (11)	
H15	0.4277	0.2692	0.3757	0.066*	
C16	0.4865 (2)	0.4291 (6)	0.3891 (2)	0.0730 (15)	
H16	0.5170	0.3989	0.3692	0.088*	
C17	0.4935 (3)	0.5485 (6)	0.4143 (2)	0.0804 (16)	
H17	0.5290	0.6035	0.4122	0.097*	
C18	0.4486 (3)	0.5877 (5)	0.4428 (2)	0.0760 (14)	
H18	0.4542	0.6708	0.4605	0.091*	
C19	0.3582 (2)	0.5207 (4)	0.68811 (16)	0.0497 (11)	
C20	0.4030 (3)	0.4923 (6)	0.7436 (2)	0.096 (2)	
H20A	0.4284	0.4134	0.7406	0.144*	
H20B	0.3775	0.4783	0.7714	0.144*	
H20C	0.4320	0.5671	0.7544	0.144*	
C21	0.1592 (2)	0.5863 (5)	0.64384 (15)	0.0496 (10)	
C22	0.1192 (3)	0.6702 (6)	0.6741 (2)	0.0832 (17)	
H22A	0.0836	0.7103	0.6475	0.125*	
H22B	0.1463	0.7394	0.6947	0.125*	
H22C	0.1020	0.6153	0.6998	0.125*	
C23	0.2282 (3)	0.4307 (6)	0.7801 (2)	0.0764 (16)	
C24	0.2089 (3)	0.3137 (6)	0.7481 (2)	0.0860 (16)	
H24A	0.2100	0.2385	0.7729	0.129*	
H24B	0.2384	0.2980	0.7236	0.129*	
H24C	0.1650	0.3250	0.7260	0.129*	
C41	0.4746 (2)	−0.0370 (5)	0.5713 (2)	0.0664 (13)	
H41A	0.4716	−0.0657	0.5332	0.100*	
H41B	0.4638	−0.1104	0.5930	0.100*	
H41C	0.5186	−0.0074	0.5870	0.100*	
C51	0.4385 (3)	0.0398 (6)	0.6760 (2)	0.0731 (14)	
H51A	0.4231	0.0802	0.7065	0.110*	
H51B	0.4856	0.0441	0.6836	0.110*	
H51C	0.4247	−0.0522	0.6721	0.110*	

### Bis(acetato-κO){5,6-dimethyl-2-(pyridin-2-yl)-1-[(pyridin-2-yl)methyl]-1H-benzimidazole-κ2N2,N3}copper(II) acetonitrile monosolvate (1) Atomic displacement parameters (Å^2^)

**Table:**

	*U*^11^	*U*^22^	*U*^33^	*U*^12^	*U*^13^	*U*^23^
Cu1	0.0377 (3)	0.0473 (3)	0.0331 (3)	−0.00463 (19)	0.0127 (2)	−0.00961 (17)
O1	0.0510 (19)	0.0698 (18)	0.0349 (13)	−0.0078 (16)	0.0135 (13)	−0.0072 (13)
O2	0.060 (2)	0.074 (2)	0.0609 (18)	−0.0027 (17)	0.0088 (16)	0.0134 (16)
O3	0.0509 (18)	0.0602 (17)	0.0513 (15)	−0.0003 (14)	0.0202 (14)	−0.0171 (13)
O4	0.062 (2)	0.064 (2)	0.073 (2)	−0.0070 (17)	0.0208 (18)	−0.0168 (16)
N1	0.0367 (17)	0.0427 (17)	0.0286 (14)	0.0010 (14)	0.0079 (12)	−0.0040 (12)
N2	0.0353 (17)	0.0401 (16)	0.0298 (14)	−0.0045 (14)	0.0110 (13)	−0.0048 (12)
N3	0.0334 (17)	0.0392 (17)	0.0432 (16)	−0.0029 (15)	0.0094 (13)	−0.0092 (13)
N4	0.054 (3)	0.060 (2)	0.065 (2)	−0.0151 (18)	0.022 (2)	−0.0073 (17)
N5	0.173 (7)	0.107 (4)	0.067 (3)	−0.016 (4)	0.048 (4)	−0.005 (3)
C1	0.037 (2)	0.040 (2)	0.0352 (17)	−0.0047 (17)	0.0099 (15)	−0.0057 (15)
C2	0.0293 (19)	0.042 (2)	0.0373 (18)	−0.0081 (16)	0.0101 (15)	−0.0053 (15)
C3	0.035 (2)	0.051 (2)	0.046 (2)	−0.0019 (18)	0.0138 (17)	−0.0086 (17)
C4	0.029 (2)	0.049 (2)	0.058 (2)	0.0010 (18)	0.0081 (17)	−0.0034 (18)
C5	0.039 (2)	0.056 (2)	0.048 (2)	−0.001 (2)	0.0043 (18)	0.0040 (18)
C6	0.039 (2)	0.054 (2)	0.0372 (18)	−0.0005 (19)	0.0087 (16)	−0.0002 (16)
C7	0.0328 (19)	0.041 (2)	0.0281 (16)	−0.0095 (16)	0.0055 (14)	−0.0062 (14)
C8	0.033 (2)	0.037 (2)	0.0366 (18)	−0.0072 (16)	0.0065 (16)	−0.0065 (14)
C9	0.052 (3)	0.045 (2)	0.0356 (19)	−0.003 (2)	0.0097 (18)	−0.0022 (16)
C10	0.058 (3)	0.047 (2)	0.045 (2)	−0.004 (2)	0.0054 (19)	0.0012 (17)
C11	0.047 (3)	0.042 (2)	0.059 (2)	0.0007 (19)	0.003 (2)	0.0033 (19)
C12	0.038 (2)	0.050 (2)	0.052 (2)	−0.0020 (19)	0.0106 (18)	−0.0107 (18)
C13	0.041 (2)	0.049 (2)	0.0284 (16)	−0.0031 (17)	0.0121 (15)	−0.0093 (14)
C14	0.040 (2)	0.053 (2)	0.0313 (17)	0.0028 (19)	0.0082 (16)	0.0042 (16)
C15	0.042 (2)	0.086 (3)	0.042 (2)	0.009 (2)	0.0181 (19)	0.011 (2)
C16	0.044 (3)	0.120 (5)	0.060 (3)	0.008 (3)	0.022 (2)	0.025 (3)
C17	0.052 (3)	0.103 (4)	0.086 (4)	−0.016 (3)	0.015 (3)	0.036 (3)
C18	0.067 (3)	0.074 (3)	0.088 (3)	−0.028 (3)	0.020 (3)	0.000 (3)
C19	0.047 (3)	0.063 (3)	0.039 (2)	0.006 (2)	0.0077 (19)	−0.0034 (18)
C20	0.081 (5)	0.134 (6)	0.059 (3)	−0.007 (4)	−0.014 (3)	0.018 (3)
C21	0.046 (2)	0.067 (3)	0.0366 (19)	0.011 (2)	0.0108 (18)	−0.0051 (19)
C22	0.073 (4)	0.103 (4)	0.083 (3)	0.018 (3)	0.039 (3)	−0.020 (3)
C23	0.103 (5)	0.081 (4)	0.050 (3)	0.003 (3)	0.026 (3)	0.011 (3)
C24	0.096 (4)	0.086 (4)	0.072 (3)	−0.008 (3)	0.010 (3)	0.017 (3)
C41	0.044 (3)	0.078 (3)	0.076 (3)	0.014 (3)	0.009 (2)	0.002 (3)
C51	0.062 (3)	0.095 (4)	0.056 (3)	0.018 (3)	0.001 (2)	0.016 (3)

### Bis(acetato-κO){5,6-dimethyl-2-(pyridin-2-yl)-1-[(pyridin-2-yl)methyl]-1H-benzimidazole-κ2N2,N3}copper(II) acetonitrile monosolvate (1) Geometric parameters (Å, º)

**Table:**

N1—C1	1.375 (5)	C8—C9	1.382 (5)
C9—C10	1.365 (6)	C10—H10	0.9500
C10—C11	1.375 (5)	C11—H11	0.9500
C11—C12	1.387 (6)	C12—H12	0.9500
N3—C12	1.323 (5)	C13—H13A	0.9900
N2—C13	1.473 (4)	C13—H13B	0.9900
C13—C14	1.517 (5)	C15—H15	0.9500
N4—C14	1.329 (5)	C16—H16	0.9500
C14—C15	1.382 (5)	C17—H17	0.9500
C15—C16	1.381 (6)	C18—H18	0.9500
C16—C17	1.361 (9)	C20—H20A	0.9800
C17—C18	1.370 (7)	C20—H20B	0.9800
N4—C18	1.324 (6)	C20—H20C	0.9800
O2—C19	1.233 (5)	C22—H22A	0.9800
O1—C19	1.271 (5)	C22—H22B	0.9800
C1—C2	1.411 (4)	C22—H22C	0.9800
N2—C2	1.388 (4)	C24—H24A	0.9800
C19—C20	1.523 (7)	C24—H24B	0.9800
O4—C21	1.224 (5)	C24—H24C	0.9800
O3—C21	1.295 (5)	C3—H3	0.9500
C21—C22	1.518 (5)	C41—H41A	0.9800
N5—C23	1.119 (7)	C41—H41B	0.9800
C23—C24	1.442 (8)	C41—H41C	0.9800
C2—C3	1.387 (5)	C51—H51A	0.9800
C3—C4	1.390 (6)	C51—H51B	0.9800
C4—C41	1.511 (6)	C51—H51C	0.9800
C4—C5	1.442 (5)	C6—H6	0.9500
C5—C51	1.526 (6)	C9—H9	0.9500
C5—C6	1.364 (6)	Cu1—N1	1.974 (3)
C1—C6	1.399 (5)	Cu1—N3	2.034 (3)
N2—C7	1.367 (4)	Cu1—O1	1.948 (3)
N1—C7	1.338 (4)	Cu1—O3	1.935 (2)
C7—C8	1.471 (5)	Cu1—O2	2.651 (3)
N3—C8	1.369 (4)	Cu1—O4	2.676 (4)
			
C1—C6—H6	120.4	C6—C1—C2	119.6 (3)
C1—N1—Cu1	137.6 (2)	C7—N2—C13	130.1 (3)
C10—C11—H11	120.8	C7—N2—C2	107.3 (3)
C10—C11—C12	118.4 (4)	C7—N1—Cu1	114.4 (2)
C10—C9—H9	120.3	C7—N1—C1	107.5 (3)
C10—C9—C8	119.4 (3)	C8—C9—H9	120.3
C11—C12—H12	118.5	C8—N3—Cu1	115.6 (2)
C11—C10—H10	120.1	C9—C10—H10	120.1
C12—C11—H11	120.8	C9—C10—C11	119.7 (4)
C12—N3—Cu1	125.8 (2)	C9—C8—C7	128.3 (3)
C12—N3—C8	118.3 (3)	H13A—C13—H13B	108.1
C14—C15—H15	120.7	H20A—C20—H20C	109.5
C14—C13—H13B	109.5	H20A—C20—H20B	109.5
C14—C13—H13A	109.5	H20B—C20—H20C	109.5
C15—C16—H16	120.6	H22A—C22—H22C	109.5
C15—C14—C13	120.1 (4)	H22A—C22—H22B	109.5
C16—C17—H17	120.7	H22B—C22—H22C	109.5
C16—C17—C18	118.7 (5)	H24A—C24—H24C	109.5
C16—C15—H15	120.7	H24A—C24—H24B	109.5
C16—C15—C14	118.6 (5)	H24B—C24—H24C	109.5
C17—C18—H18	117.9	H41A—C41—H41C	109.5
C17—C16—H16	120.6	H41A—C41—H41B	109.5
C17—C16—C15	118.7 (4)	H41B—C41—H41C	109.5
C18—C17—H17	120.7	H51A—C51—H51C	109.5
C18—N4—C14	116.9 (4)	H51A—C51—H51B	109.5
C19—C20—H20C	109.5	H51B—C51—H51C	109.5
C19—C20—H20B	109.5	N1—C7—C8	118.4 (3)
C19—C20—H20A	109.5	N1—C7—N2	110.9 (3)
C19—O1—Cu1	106.7 (2)	N1—C1—C2	108.2 (3)
C2—C3—H3	120.6	N1—C1—C6	132.2 (3)
C2—C3—C4	118.7 (3)	N1—Cu1—N3	80.41 (11)
C2—N2—C13	122.0 (3)	N2—C13—H13B	109.5
C21—C22—H22C	109.5	N2—C13—H13A	109.5
C21—C22—H22B	109.5	N2—C13—C14	110.8 (3)
C21—C22—H22A	109.5	N2—C7—C8	130.7 (3)
C21—O3—Cu1	107.4 (2)	N2—C2—C1	106.2 (3)
C23—C24—H24C	109.5	N3—C12—H12	118.5
C23—C24—H24B	109.5	N3—C12—C11	122.9 (3)
C23—C24—H24A	109.5	N3—C8—C7	110.5 (3)
C3—C4—C41	119.6 (3)	N3—C8—C9	121.2 (3)
C3—C4—C5	119.4 (3)	N4—C18—H18	117.9
C3—C2—C1	121.7 (3)	N4—C18—C17	124.2 (5)
C3—C2—N2	132.2 (3)	N4—C14—C13	116.9 (3)
C4—C41—H41C	109.5	N4—C14—C15	122.9 (4)
C4—C41—H41B	109.5	N5—C23—C24	179.5 (9)
C4—C41—H41A	109.5	O1—C19—C20	115.7 (4)
C4—C5—C51	119.8 (4)	O1—Cu1—N3	171.88 (11)
C4—C3—H3	120.6	O1—Cu1—N1	94.59 (12)
C5—C51—H51C	109.5	O2—C19—C20	120.7 (5)
C5—C51—H51B	109.5	O2—C19—O1	123.7 (4)
C5—C51—H51A	109.5	O3—C21—C22	114.5 (4)
C5—C6—H6	120.4	O3—Cu1—N3	93.35 (12)
C5—C6—C1	119.3 (3)	O3—Cu1—N1	173.39 (12)
C5—C4—C41	120.9 (4)	O3—Cu1—O1	91.85 (12)
C6—C5—C51	119.0 (4)	O4—C21—C22	122.4 (4)
C6—C5—C4	121.2 (4)	O4—C21—O3	123.2 (3)
			
C7—N1—C1—C6	−179.0 (4)	C12—N3—C8—C9	0.6 (5)
Cu1—N1—C1—C6	10.3 (6)	Cu1—N3—C8—C9	−172.7 (3)
C7—N1—C1—C2	−0.5 (4)	C12—N3—C8—C7	179.7 (3)
Cu1—N1—C1—C2	−171.2 (3)	Cu1—N3—C8—C7	6.4 (4)
C7—N2—C2—C3	−178.6 (4)	N1—C7—C8—N3	0.0 (5)
C13—N2—C2—C3	−6.6 (6)	N2—C7—C8—N3	178.5 (3)
C7—N2—C2—C1	1.7 (4)	N1—C7—C8—C9	179.0 (4)
C13—N2—C2—C1	173.6 (3)	N2—C7—C8—C9	−2.5 (6)
N1—C1—C2—C3	179.4 (3)	N3—C8—C9—C10	−0.7 (6)
C6—C1—C2—C3	−1.9 (5)	C7—C8—C9—C10	−179.5 (4)
N1—C1—C2—N2	−0.8 (4)	C8—C9—C10—C11	0.0 (6)
C6—C1—C2—N2	178.0 (3)	C9—C10—C11—C12	0.6 (6)
N2—C2—C3—C4	−179.7 (4)	C8—N3—C12—C11	0.0 (6)
C1—C2—C3—C4	0.1 (5)	Cu1—N3—C12—C11	172.6 (3)
C2—C3—C4—C5	1.2 (5)	C10—C11—C12—N3	−0.7 (6)
C2—C3—C4—C41	179.5 (4)	C7—N2—C13—C14	88.6 (4)
C3—C4—C5—C6	−0.7 (6)	C2—N2—C13—C14	−81.3 (4)
C41—C4—C5—C6	−179.0 (4)	C18—N4—C14—C15	−0.1 (7)
C3—C4—C5—C51	177.8 (4)	C18—N4—C14—C13	−179.9 (4)
C41—C4—C5—C51	−0.6 (6)	N2—C13—C14—N4	−44.2 (5)
C4—C5—C6—C1	−1.1 (6)	N2—C13—C14—C15	136.1 (4)
C51—C5—C6—C1	−179.6 (4)	N4—C14—C15—C16	0.0 (6)
N1—C1—C6—C5	−179.3 (4)	C13—C14—C15—C16	179.8 (4)
C2—C1—C6—C5	2.3 (5)	C14—C15—C16—C17	−0.2 (7)
C1—N1—C7—N2	1.6 (4)	C15—C16—C17—C18	0.5 (8)
Cu1—N1—C7—N2	174.7 (2)	C14—N4—C18—C17	0.4 (8)
C1—N1—C7—C8	−179.6 (3)	C16—C17—C18—N4	−0.6 (9)
Cu1—N1—C7—C8	−6.5 (4)	Cu1—O1—C19—O2	−1.9 (5)
C2—N2—C7—N1	−2.1 (4)	Cu1—O1—C19—C20	179.1 (4)
C13—N2—C7—N1	−173.1 (3)	Cu1—O3—C21—O4	−5.5 (5)
C2—N2—C7—C8	179.4 (3)	Cu1—O3—C21—C22	174.7 (3)
C13—N2—C7—C8	8.3 (6)		

### Bis(acetato-κO){5,6-dimethyl-2-(pyridin-2-yl)-1-[(pyridin-2-yl)methyl]-1H-benzimidazole-κ2N2,N3}copper(II) acetonitrile monosolvate (1) Hydrogen-bond geometry (Å, º)

*Cg*(Bz) is the centroid of the benzene ring.

**Table:**

*D*—H···*A*	*D*—H	H···*A*	*D*···*A*	*D*—H···*A*
C12—H12···O3	0.95	2.51	3.043 (5)	115
C10—H10···O2^i^	0.95	2.42	3.140 (5)	132
C6—H6···O1	0.95	2.54	3.191 (5)	126
C24—H24*B*···O1	0.98	2.59	3.334 (6)	133
C9—H9···N5^ii^	0.95	2.50	3.349 (6)	149
C13—H13*B*···O4^iii^	0.99	2.41	3.391 (5)	169
C16—H16···O2^iv^	0.95	2.57	3.449 (6)	154
C17—H17···*Cg*(Bz)^v^	0.95	2.87	3.783 (6)	162

Symmetry codes: (i) −*x*+1/2, −*y*+3/2, −*z*+1; (ii) *x*, −*y*+1, *z*−1/2; (iii) −*x*+1/2, −*y*+1/2, −*z*+1; (iv) −*x*+1, −*y*+1, −*z*+1; (v) *x*+3/2, *y*+3/2, *z*+1.

### Bis(acetato-κO){5,6-dimethyl-2-(pyridin-2-yl)-1-[pyridin-2-yl)methyl]-1H-benzimidazole-κ2N2,N3}copper(II) ethanol hemisolvate (2) Crystal data

**Table:**

[Cu(C_2_H_3_O_2_)_2_(C_20_H_18_N_4_)]·0.5C_2_H_6_O	*Z* = 2
*M**_r_* = 1038.09	*F*(000) = 1080
Triclinic, *P*1	*D*_x_ = 1.386 Mg m^−^^3^
*a* = 11.2595 (11) Å	Mo *K*α radiation, λ = 0.71073 Å
*b* = 14.0130 (15) Å	Cell parameters from 7750 reflections
*c* = 16.5004 (18) Å	θ = 2.3–23.9°
α = 81.873 (4)°	µ = 0.92 mm^−^^1^
β = 77.126 (4)°	*T* = 200 K
γ = 80.348 (4)°	Parallelpiped, blue-green
*V* = 2487.4 (5) Å^3^	0.60 × 0.30 × 0.20 mm

### Bis(acetato-κO){5,6-dimethyl-2-(pyridin-2-yl)-1-[pyridin-2-yl)methyl]-1H-benzimidazole-κ2N2,N3}copper(II) ethanol hemisolvate (2) Data collection

**Table:**

Bruker SMART X2S benchtop diffractometer	9149 independent reflections
Radiation source: sealed microfocus tube	6690 reflections with *I* > 2σ(*I*)
Detector resolution: 8.3330 pixels mm^-1^	*R*_int_ = 0.042
ω scans	θ_max_ = 25.7°, θ_min_ = 1.3°
Absorption correction: multi-scan (SADABS; Bruker, 2013)	*h* = −13→13
*T*_min_ = 0.726, *T*_max_ = 0.832	*k* = −17→16
22519 measured reflections	*l* = −20→15

### Bis(acetato-κO){5,6-dimethyl-2-(pyridin-2-yl)-1-[pyridin-2-yl)methyl]-1H-benzimidazole-κ2N2,N3}copper(II) ethanol hemisolvate (2) Refinement

**Table:**

Refinement on *F*^2^	160 restraints
Least-squares matrix: full	Hydrogen site location: inferred from neighbouring sites
*R*[*F*^2^ > 2σ(*F*^2^)] = 0.045	H-atom parameters constrained
*wR*(*F*^2^) = 0.121	*w* = 1/[σ^2^(*F*_o_^2^) + (0.0537*P*)^2^ + 0.9243*P*] where *P* = (*F*_o_^2^ + 2*F*_c_^2^)/3
*S* = 1.04	(Δ/σ)_max_ = 0.001
9149 reflections	Δρ_max_ = 0.77 e Å^−^^3^
690 parameters	Δρ_min_ = −0.42 e Å^−^^3^

### Bis(acetato-κO){5,6-dimethyl-2-(pyridin-2-yl)-1-[pyridin-2-yl)methyl]-1H-benzimidazole-κ2N2,N3}copper(II) ethanol hemisolvate (2) Special details

**Table:**

Geometry. All esds (except the esd in the dihedral angle between two l.s. planes) are estimated using the full covariance matrix. The cell esds are taken into account individually in the estimation of esds in distances, angles and torsion angles; correlations between esds in cell parameters are only used when they are defined by crystal symmetry. An approximate (isotropic) treatment of cell esds is used for estimating esds involving l.s. planes.
Refinement. Crystal data, data collection and structure refinement details are summarized in Table 1. Early in the refinement of (2), the ethanol hemisolvate molecule was found to be disordered. The disorder was modeled using three contributors. Successful refinement required the use of O–H, C–C and C–O distance restraints of 0.84 Å, 1.53 Å and 1.43 Å, respectively, and restraints on the *U^ij^* components of the anisotropically refined atoms in the disordered ethanol. The disorder model refined to occupancies of 0.411 (3) : 0.362 (3) : 0.227 (3). All H atoms were located in difference Fourier maps, except those associated with the disordered ethanol molecule. H atoms bonded to C atoms were refined using a riding model, with C–H = 0.95 Å and *U*_iso_(H) = 1.2*U*_eq_(C) for the aromatic positions; C–H = 0.99 Å and *U*_iso_(H) = 1.2*U*_eq_(C) for the methylene groups; and C–H = 0.98 Å and *U*_iso_(H) = 1.5*U*_eq_(C) for the methyl groups. The hydroxy H atoms in the disordered ethanol contributors were refined using a rotating group model with C–O–H tetrahedral, distance restraints to acceptor atoms (O6 and symmetry-generated O2) and with *U*_iso_(H) = 1.5*U*_eq_(O).

### Bis(acetato-κO){5,6-dimethyl-2-(pyridin-2-yl)-1-[pyridin-2-yl)methyl]-1H-benzimidazole-κ2N2,N3}copper(II) ethanol hemisolvate (2) Fractional atomic coordinates and isotropic or equivalent isotropic displacement parameters (Å^2^)

**Table:**

	*x*	*y*	*z*	*U*_iso_*/*U*_eq_	Occ. (<1)
C110	0.7322 (12)	0.7207 (12)	0.2553 (10)	0.112 (3)	0.411 (3)
H111	0.6863	0.6731	0.2425	0.169*	0.411 (3)
H112	0.6784	0.7611	0.2965	0.169*	0.411 (3)
H113	0.7611	0.7622	0.2040	0.169*	0.411 (3)
C100	0.8389 (12)	0.6691 (9)	0.2895 (11)	0.122 (3)	0.411 (3)
H101	0.8086	0.6261	0.3403	0.147*	0.411 (3)
H102	0.8911	0.6272	0.2479	0.147*	0.411 (3)
O100	0.9097 (8)	0.7285 (6)	0.3096 (5)	0.112 (3)	0.411 (3)
H100	0.8830	0.7405	0.3594	0.168*	0.411 (3)
C210	0.6049 (12)	0.6081 (10)	0.2736 (10)	0.114 (3)	0.362 (3)
H211	0.5941	0.5404	0.2942	0.171*	0.362 (3)
H212	0.5726	0.6492	0.3192	0.171*	0.362 (3)
H213	0.5604	0.6305	0.2281	0.171*	0.362 (3)
C200	0.7342 (13)	0.6145 (9)	0.2429 (12)	0.111 (3)	0.362 (3)
H201	0.7788	0.5903	0.2888	0.134*	0.362 (3)
H202	0.7666	0.5716	0.1977	0.134*	0.362 (3)
O200	0.7576 (11)	0.7075 (9)	0.2126 (8)	0.123 (3)	0.362 (3)
H200	0.6907	0.7445	0.2123	0.184*	0.362 (3)
C310	0.825 (2)	0.7134 (19)	0.2390 (18)	0.119 (3)	0.227 (3)
H311	0.8604	0.7241	0.2857	0.178*	0.227 (3)
H312	0.8911	0.6861	0.1952	0.178*	0.227 (3)
H313	0.7846	0.7755	0.2162	0.178*	0.227 (3)
C300	0.731 (2)	0.642 (2)	0.2700 (12)	0.117 (3)	0.227 (3)
H301	0.7704	0.5785	0.2927	0.140*	0.227 (3)
H302	0.6631	0.6685	0.3138	0.140*	0.227 (3)
O300	0.6892 (17)	0.6336 (13)	0.1987 (12)	0.119 (3)	0.227 (3)
H300	0.6256	0.6740	0.1962	0.178*	0.227 (3)
Cu1	1.12110 (3)	0.24202 (3)	0.42497 (2)	0.03483 (12)	
Cu2	0.39897 (4)	0.74583 (3)	0.09205 (2)	0.03675 (13)	
O1	1.2626 (2)	0.30596 (16)	0.41826 (15)	0.0443 (6)	
O2	1.2708 (3)	0.2118 (2)	0.53576 (19)	0.0743 (9)	
O3	1.0065 (2)	0.33510 (17)	0.49500 (15)	0.0465 (6)	
O4	0.99188 (19)	0.38173 (17)	0.36382 (15)	0.0439 (6)	
O5	0.4991 (2)	0.83679 (16)	0.01689 (15)	0.0495 (6)	
O6	0.5495 (3)	0.8340 (2)	0.13825 (16)	0.0767 (10)	
O7	0.2561 (2)	0.84483 (16)	0.11952 (16)	0.0513 (6)	
O8	0.2187 (2)	0.77443 (17)	0.01778 (15)	0.0518 (6)	
N1	1.2083 (2)	0.15678 (18)	0.33409 (16)	0.0341 (6)	
N2	1.0026 (2)	0.15012 (18)	0.43873 (15)	0.0335 (6)	
N3	0.9448 (2)	0.02312 (18)	0.39860 (17)	0.0366 (6)	
N4	0.9083 (2)	0.06519 (19)	0.23244 (17)	0.0403 (7)	
N5	0.3178 (2)	0.64194 (19)	0.17383 (16)	0.0359 (6)	
N6	0.5232 (2)	0.63011 (18)	0.06515 (15)	0.0318 (6)	
N7	0.5810 (2)	0.46992 (18)	0.08607 (16)	0.0326 (6)	
N8	0.5714 (2)	0.26486 (19)	0.24464 (17)	0.0416 (7)	
C1	1.1481 (3)	0.0844 (2)	0.3236 (2)	0.0349 (7)	
C2	1.1963 (3)	0.0241 (2)	0.2612 (2)	0.0444 (8)	
H2	1.1531	−0.0259	0.2541	0.053*	
C3	1.3089 (3)	0.0375 (3)	0.2089 (2)	0.0518 (9)	
H3	1.3448	−0.0043	0.1667	0.062*	
C4	1.3676 (3)	0.1124 (3)	0.2192 (2)	0.0509 (9)	
H4	1.4433	0.1243	0.1831	0.061*	
C5	1.3151 (3)	0.1697 (3)	0.2825 (2)	0.0442 (8)	
H5	1.3565	0.2206	0.2897	0.053*	
C6	1.0319 (3)	0.0831 (2)	0.38545 (19)	0.0333 (7)	
C7	0.8886 (3)	0.1354 (2)	0.48849 (19)	0.0353 (7)	
C8	0.8512 (3)	0.0567 (2)	0.4630 (2)	0.0374 (8)	
C9	0.7376 (3)	0.0263 (3)	0.4987 (2)	0.0476 (9)	
H9	0.7130	−0.0275	0.4806	0.057*	
C10	0.6623 (3)	0.0768 (3)	0.5608 (2)	0.0512 (10)	
C11	0.6995 (3)	0.1566 (3)	0.5885 (2)	0.0497 (9)	
C12	0.8135 (3)	0.1856 (3)	0.5526 (2)	0.0420 (8)	
H12	0.8395	0.2383	0.5713	0.050*	
C13	0.9321 (3)	−0.0540 (2)	0.3508 (2)	0.0425 (8)	
H13A	0.8857	−0.1027	0.3884	0.051*	
H13B	1.0146	−0.0873	0.3269	0.051*	
C14	0.8655 (3)	−0.0113 (2)	0.2814 (2)	0.0365 (7)	
C15	0.7647 (3)	−0.0491 (3)	0.2714 (2)	0.0433 (8)	
H15	0.7362	−0.1029	0.3081	0.052*	
C16	0.7061 (3)	−0.0076 (3)	0.2074 (2)	0.0500 (9)	
H16	0.6372	−0.0327	0.1988	0.060*	
C17	0.7491 (3)	0.0709 (3)	0.1563 (2)	0.0454 (9)	
H17	0.7102	0.1014	0.1119	0.054*	
C18	0.8502 (3)	0.1044 (2)	0.1710 (2)	0.0434 (8)	
H18	0.8800	0.1583	0.1352	0.052*	
C19	0.9574 (3)	0.3905 (2)	0.4395 (2)	0.0416 (8)	
C20	0.8523 (4)	0.4683 (3)	0.4697 (3)	0.0672 (12)	
H20A	0.7965	0.4822	0.4303	0.101*	
H20B	0.8852	0.5278	0.4731	0.101*	
H20C	0.8074	0.4454	0.5250	0.101*	
C21	1.3077 (3)	0.2774 (3)	0.4830 (2)	0.0435 (8)	
C22	1.4111 (3)	0.3294 (3)	0.4922 (3)	0.0648 (12)	
H22A	1.3859	0.3626	0.5430	0.097*	
H22B	1.4295	0.3773	0.4435	0.097*	
H22C	1.4846	0.2818	0.4960	0.097*	
C23	0.6360 (3)	0.6074 (2)	0.01201 (18)	0.0317 (7)	
C24	0.6730 (3)	0.5073 (2)	0.02474 (19)	0.0331 (7)	
C25	0.7842 (3)	0.4623 (2)	−0.0185 (2)	0.0396 (8)	
H25	0.8078	0.3939	−0.0096	0.047*	
C26	0.8592 (3)	0.5209 (3)	−0.0748 (2)	0.0420 (8)	
C27	0.8222 (3)	0.6228 (3)	−0.0891 (2)	0.0423 (8)	
C28	0.7099 (3)	0.6658 (2)	−0.04656 (19)	0.0375 (8)	
H28	0.6840	0.7337	−0.0571	0.045*	
C29	0.4942 (3)	0.5477 (2)	0.10875 (18)	0.0321 (7)	
C30	0.3812 (3)	0.5508 (2)	0.17337 (19)	0.0332 (7)	
C31	0.3395 (3)	0.4751 (3)	0.2297 (2)	0.0487 (9)	
H31	0.3863	0.4120	0.2300	0.058*	
C32	0.2293 (3)	0.4920 (3)	0.2857 (3)	0.0627 (12)	
H32	0.1988	0.4405	0.3244	0.075*	
C33	0.1643 (3)	0.5840 (3)	0.2849 (2)	0.0600 (11)	
H33	0.0877	0.5969	0.3225	0.072*	
C34	0.2117 (3)	0.6574 (3)	0.2286 (2)	0.0482 (9)	
H34	0.1673	0.7213	0.2289	0.058*	
C35	0.5817 (3)	0.3659 (2)	0.1147 (2)	0.0367 (7)	
H35A	0.6344	0.3279	0.0704	0.044*	
H35B	0.4971	0.3502	0.1228	0.044*	
C36	0.6272 (3)	0.3344 (2)	0.19473 (19)	0.0318 (7)	
C37	0.7230 (3)	0.3699 (3)	0.2131 (2)	0.0530 (10)	
H37	0.7610	0.4195	0.1760	0.064*	
C38	0.7643 (3)	0.3336 (3)	0.2854 (2)	0.0591 (11)	
H38	0.8303	0.3579	0.2992	0.071*	
C39	0.7086 (4)	0.2624 (3)	0.3365 (2)	0.0577 (11)	
H39	0.7348	0.2356	0.3868	0.069*	
C40	0.6135 (4)	0.2297 (3)	0.3140 (2)	0.0561 (10)	
H40	0.5755	0.1792	0.3498	0.067*	
C41	0.5640 (4)	0.8600 (3)	0.0627 (2)	0.0557 (11)	
C42	0.6635 (4)	0.9222 (3)	0.0194 (3)	0.0776 (15)	
H42A	0.6269	0.9908	0.0137	0.116*	
H42B	0.7006	0.9016	−0.0361	0.116*	
H42C	0.7270	0.9143	0.0529	0.116*	
C43	0.1881 (3)	0.8350 (2)	0.0695 (2)	0.0480 (9)	
C44	0.0649 (4)	0.8991 (3)	0.0772 (3)	0.0868 (16)	
H44A	0.0259	0.8910	0.0317	0.130*	
H44B	0.0769	0.9673	0.0740	0.130*	
H44C	0.0121	0.8807	0.1310	0.130*	
C45	0.6167 (4)	0.2099 (3)	0.6582 (2)	0.0688 (12)	
H45A	0.6094	0.1664	0.7105	0.103*	
H45B	0.5351	0.2306	0.6448	0.103*	
H45C	0.6520	0.2671	0.6644	0.103*	
C46	0.5361 (3)	0.0466 (3)	0.5996 (3)	0.0733 (14)	
H46A	0.5284	−0.0131	0.5778	0.110*	
H46B	0.4718	0.0986	0.5855	0.110*	
H46C	0.5269	0.0349	0.6605	0.110*	
C47	0.9831 (3)	0.4749 (3)	−0.1201 (3)	0.0614 (11)	
H47A	1.0486	0.4963	−0.0998	0.092*	
H47B	0.9921	0.4947	−0.1803	0.092*	
H47C	0.9888	0.4038	−0.1096	0.092*	
C48	0.9058 (3)	0.6858 (3)	−0.1512 (2)	0.0589 (11)	
H48A	0.8701	0.7544	−0.1494	0.088*	
H48B	0.9142	0.6677	−0.2076	0.088*	
H48C	0.9870	0.6759	−0.1365	0.088*	

### Bis(acetato-κO){5,6-dimethyl-2-(pyridin-2-yl)-1-[pyridin-2-yl)methyl]-1H-benzimidazole-κ2N2,N3}copper(II) ethanol hemisolvate (2) Atomic displacement parameters (Å^2^)

**Table:**

	*U*^11^	*U*^22^	*U*^33^	*U*^12^	*U*^13^	*U*^23^
C110	0.135 (6)	0.093 (5)	0.107 (6)	0.021 (5)	−0.026 (6)	−0.047 (5)
C100	0.138 (6)	0.095 (5)	0.113 (6)	0.042 (5)	−0.014 (5)	−0.031 (5)
O100	0.145 (6)	0.058 (4)	0.094 (5)	0.043 (4)	0.022 (5)	−0.018 (4)
C210	0.137 (7)	0.092 (6)	0.115 (7)	0.032 (6)	−0.029 (6)	−0.067 (6)
C200	0.131 (5)	0.087 (5)	0.109 (6)	0.034 (5)	−0.024 (5)	−0.046 (5)
O200	0.133 (6)	0.103 (5)	0.128 (6)	0.035 (5)	−0.038 (5)	−0.044 (5)
C310	0.138 (6)	0.085 (5)	0.114 (7)	0.044 (6)	−0.013 (6)	−0.038 (5)
C300	0.133 (6)	0.092 (5)	0.112 (6)	0.038 (5)	−0.020 (5)	−0.040 (5)
O300	0.136 (6)	0.090 (6)	0.124 (7)	0.025 (5)	−0.027 (6)	−0.042 (5)
Cu1	0.0377 (2)	0.0332 (2)	0.0358 (2)	−0.01181 (17)	−0.01069 (18)	0.00191 (17)
Cu2	0.0470 (3)	0.0298 (2)	0.0306 (2)	−0.00976 (18)	0.00089 (18)	−0.00279 (16)
O1	0.0466 (14)	0.0423 (13)	0.0491 (14)	−0.0183 (11)	−0.0165 (12)	0.0033 (11)
O2	0.0743 (19)	0.076 (2)	0.074 (2)	−0.0280 (16)	−0.0333 (16)	0.0363 (17)
O3	0.0547 (15)	0.0452 (14)	0.0430 (14)	−0.0137 (12)	−0.0092 (12)	−0.0094 (12)
O4	0.0373 (13)	0.0484 (14)	0.0458 (14)	−0.0078 (11)	−0.0075 (11)	−0.0035 (12)
O5	0.0639 (16)	0.0388 (13)	0.0432 (14)	−0.0180 (12)	0.0016 (12)	−0.0031 (11)
O6	0.101 (2)	0.095 (2)	0.0399 (16)	−0.0591 (19)	0.0092 (16)	−0.0094 (15)
O7	0.0645 (17)	0.0332 (13)	0.0486 (15)	−0.0038 (12)	0.0041 (13)	−0.0077 (11)
O8	0.0626 (16)	0.0402 (14)	0.0452 (14)	0.0069 (12)	−0.0052 (12)	−0.0058 (12)
N1	0.0306 (15)	0.0316 (14)	0.0389 (15)	−0.0049 (11)	−0.0088 (12)	0.0038 (12)
N2	0.0367 (16)	0.0330 (14)	0.0321 (14)	−0.0109 (12)	−0.0111 (12)	0.0055 (11)
N3	0.0406 (16)	0.0286 (14)	0.0448 (16)	−0.0117 (12)	−0.0201 (13)	0.0079 (12)
N4	0.0445 (17)	0.0312 (15)	0.0472 (17)	−0.0083 (12)	−0.0143 (14)	0.0008 (13)
N5	0.0350 (15)	0.0386 (15)	0.0313 (14)	−0.0086 (12)	−0.0008 (12)	−0.0001 (12)
N6	0.0335 (15)	0.0326 (14)	0.0293 (13)	−0.0117 (11)	−0.0039 (12)	0.0010 (11)
N7	0.0357 (15)	0.0294 (14)	0.0341 (14)	−0.0085 (12)	−0.0117 (12)	0.0041 (11)
N8	0.0444 (17)	0.0370 (16)	0.0406 (16)	−0.0079 (13)	−0.0072 (13)	0.0055 (13)
C1	0.0343 (18)	0.0301 (16)	0.0406 (18)	−0.0035 (14)	−0.0135 (15)	0.0030 (14)
C2	0.047 (2)	0.0352 (18)	0.052 (2)	−0.0029 (15)	−0.0152 (18)	−0.0050 (16)
C3	0.047 (2)	0.052 (2)	0.052 (2)	0.0073 (18)	−0.0094 (19)	−0.0088 (18)
C4	0.036 (2)	0.055 (2)	0.054 (2)	−0.0020 (17)	−0.0022 (18)	0.0020 (19)
C5	0.034 (2)	0.046 (2)	0.050 (2)	−0.0080 (16)	−0.0066 (17)	0.0038 (17)
C6	0.0362 (18)	0.0306 (16)	0.0354 (17)	−0.0089 (14)	−0.0157 (15)	0.0063 (14)
C7	0.0343 (19)	0.0386 (18)	0.0333 (17)	−0.0116 (14)	−0.0130 (15)	0.0121 (14)
C8	0.0362 (19)	0.0416 (19)	0.0356 (18)	−0.0126 (15)	−0.0155 (16)	0.0131 (15)
C9	0.042 (2)	0.053 (2)	0.051 (2)	−0.0215 (18)	−0.0221 (18)	0.0220 (18)
C10	0.037 (2)	0.070 (3)	0.044 (2)	−0.0187 (19)	−0.0142 (17)	0.028 (2)
C11	0.040 (2)	0.069 (3)	0.0342 (19)	−0.0053 (19)	−0.0080 (16)	0.0147 (18)
C12	0.041 (2)	0.050 (2)	0.0340 (18)	−0.0107 (16)	−0.0104 (16)	0.0077 (16)
C13	0.049 (2)	0.0318 (17)	0.052 (2)	−0.0162 (15)	−0.0186 (17)	0.0017 (16)
C14	0.0394 (19)	0.0316 (17)	0.0415 (19)	−0.0048 (14)	−0.0148 (15)	−0.0031 (15)
C15	0.046 (2)	0.043 (2)	0.043 (2)	−0.0144 (16)	−0.0115 (17)	−0.0012 (16)
C16	0.040 (2)	0.066 (3)	0.048 (2)	−0.0141 (18)	−0.0115 (17)	−0.012 (2)
C17	0.046 (2)	0.053 (2)	0.0366 (19)	0.0000 (17)	−0.0106 (16)	−0.0070 (17)
C18	0.050 (2)	0.0340 (18)	0.043 (2)	−0.0043 (16)	−0.0065 (17)	−0.0008 (16)
C19	0.036 (2)	0.0383 (19)	0.053 (2)	−0.0116 (15)	−0.0047 (18)	−0.0126 (18)
C20	0.057 (3)	0.058 (3)	0.084 (3)	0.004 (2)	−0.005 (2)	−0.027 (2)
C21	0.038 (2)	0.041 (2)	0.053 (2)	−0.0035 (16)	−0.0156 (17)	−0.0039 (17)
C22	0.047 (2)	0.079 (3)	0.079 (3)	−0.019 (2)	−0.022 (2)	−0.018 (2)
C23	0.0312 (18)	0.0363 (17)	0.0293 (16)	−0.0101 (14)	−0.0069 (14)	−0.0017 (13)
C24	0.0339 (18)	0.0366 (18)	0.0318 (16)	−0.0108 (14)	−0.0100 (14)	−0.0010 (14)
C25	0.040 (2)	0.0364 (18)	0.0432 (19)	−0.0028 (15)	−0.0123 (16)	−0.0050 (16)
C26	0.0350 (19)	0.053 (2)	0.0399 (19)	−0.0054 (16)	−0.0072 (16)	−0.0122 (17)
C27	0.042 (2)	0.051 (2)	0.0336 (18)	−0.0144 (17)	−0.0033 (16)	−0.0038 (16)
C28	0.0391 (19)	0.0361 (18)	0.0356 (18)	−0.0106 (15)	−0.0015 (15)	−0.0013 (15)
C29	0.0322 (18)	0.0342 (17)	0.0317 (16)	−0.0084 (14)	−0.0111 (14)	0.0024 (14)
C30	0.0283 (17)	0.0383 (18)	0.0341 (17)	−0.0106 (14)	−0.0102 (14)	0.0059 (14)
C31	0.031 (2)	0.045 (2)	0.062 (2)	−0.0077 (16)	−0.0063 (18)	0.0198 (18)
C32	0.041 (2)	0.064 (3)	0.069 (3)	−0.011 (2)	−0.003 (2)	0.030 (2)
C33	0.038 (2)	0.071 (3)	0.056 (2)	−0.008 (2)	0.0071 (19)	0.017 (2)
C34	0.046 (2)	0.045 (2)	0.045 (2)	−0.0034 (17)	−0.0012 (18)	0.0064 (17)
C35	0.0369 (18)	0.0292 (16)	0.0469 (19)	−0.0101 (14)	−0.0149 (15)	0.0024 (14)
C36	0.0258 (16)	0.0335 (17)	0.0343 (17)	−0.0026 (13)	−0.0067 (14)	0.0012 (14)
C37	0.044 (2)	0.073 (3)	0.047 (2)	−0.0241 (19)	−0.0187 (17)	0.0141 (19)
C38	0.037 (2)	0.094 (3)	0.048 (2)	−0.011 (2)	−0.0174 (18)	−0.001 (2)
C39	0.054 (2)	0.071 (3)	0.039 (2)	0.016 (2)	−0.0157 (19)	0.003 (2)
C40	0.066 (3)	0.052 (2)	0.044 (2)	−0.010 (2)	−0.010 (2)	0.0138 (18)
C41	0.077 (3)	0.045 (2)	0.043 (2)	−0.028 (2)	0.010 (2)	−0.0126 (18)
C42	0.108 (4)	0.077 (3)	0.053 (2)	−0.063 (3)	0.014 (3)	−0.014 (2)
C43	0.058 (2)	0.0282 (18)	0.043 (2)	0.0048 (16)	0.0064 (19)	0.0054 (16)
C44	0.078 (3)	0.078 (3)	0.082 (3)	0.037 (3)	−0.005 (3)	−0.009 (3)
C45	0.051 (2)	0.100 (4)	0.047 (2)	−0.010 (2)	−0.001 (2)	0.006 (2)
C46	0.042 (2)	0.101 (4)	0.069 (3)	−0.027 (2)	−0.012 (2)	0.035 (3)
C47	0.044 (2)	0.069 (3)	0.065 (3)	0.000 (2)	0.003 (2)	−0.015 (2)
C48	0.051 (2)	0.066 (3)	0.052 (2)	−0.017 (2)	0.0100 (19)	−0.001 (2)

### Bis(acetato-κO){5,6-dimethyl-2-(pyridin-2-yl)-1-[pyridin-2-yl)methyl]-1H-benzimidazole-κ2N2,N3}copper(II) ethanol hemisolvate (2) Geometric parameters (Å, º)

**Table:**

N1—C1	1.361 (4)	C12—H12	0.9500
C9—C10	1.373 (5)	C13—H13A	0.9900
C110—C100	1.476 (9)	C13—H13B	0.9900
C10—C11	1.423 (5)	C15—H15	0.9500
C11—C12	1.390 (5)	C16—H16	0.9500
C7—C12	1.393 (4)	C17—H17	0.9500
N3—C13	1.466 (4)	C18—H18	0.9500
C13—C14	1.507 (5)	C2—H2	0.9500
N4—C14	1.337 (4)	O200—H200	0.8400
C14—C15	1.381 (4)	C200—H201	0.9900
C15—C16	1.377 (5)	C200—H202	0.9900
C16—C17	1.375 (5)	C20—H20A	0.9800
C17—C18	1.382 (5)	C20—H20B	0.9800
N4—C18	1.332 (4)	C20—H20C	0.9800
O4—C19	1.240 (4)	C210—H211	0.9800
O3—C19	1.275 (4)	C210—H212	0.9800
Cu1—C19	2.538 (3)	C210—H213	0.9800
C1—C2	1.383 (4)	C22—H22A	0.9800
C19—C20	1.515 (5)	C22—H22B	0.9800
C210—C200	1.444 (9)	C22—H22C	0.9800
O2—C21	1.232 (4)	C25—H25	0.9500
O1—C21	1.269 (4)	C28—H28	0.9500
C21—C22	1.517 (5)	C3—H3	0.9500
N6—C23	1.389 (4)	O300—H300	0.8400
C23—C24	1.394 (4)	C300—H301	0.9900
N7—C24	1.392 (4)	C300—H302	0.9900
C24—C25	1.393 (4)	C31—H31	0.9500
C25—C26	1.385 (4)	C310—H311	0.9800
C26—C27	1.420 (5)	C310—H312	0.9800
C27—C28	1.387 (4)	C310—H313	0.9800
C23—C28	1.395 (4)	C32—H32	0.9500
N7—C29	1.370 (4)	C33—H33	0.9500
N6—C29	1.321 (4)	C34—H34	0.9500
C2—C3	1.390 (5)	C35—H35A	0.9900
C29—C30	1.466 (4)	C35—H35B	0.9900
N5—C30	1.355 (4)	C37—H37	0.9500
C310—C300	1.532 (10)	C38—H38	0.9500
C30—C31	1.379 (4)	C39—H39	0.9500
C31—C32	1.379 (5)	C4—H4	0.9500
C32—C33	1.372 (5)	C40—H40	0.9500
C33—C34	1.377 (5)	C42—H42A	0.9800
N5—C34	1.333 (4)	C42—H42B	0.9800
N7—C35	1.467 (4)	C42—H42C	0.9800
C35—C36	1.504 (4)	C44—H44A	0.9800
N8—C36	1.330 (4)	C44—H44B	0.9800
C36—C37	1.368 (4)	C44—H44C	0.9800
C37—C38	1.377 (5)	C45—H45A	0.9800
C38—C39	1.355 (5)	C45—H45B	0.9800
C3—C4	1.380 (5)	C45—H45C	0.9800
C39—C40	1.373 (5)	C46—H46A	0.9800
N8—C40	1.334 (5)	C46—H46B	0.9800
O6—C41	1.231 (4)	C46—H46C	0.9800
O5—C41	1.267 (4)	C47—H47A	0.9800
C41—C42	1.523 (5)	C47—H47B	0.9800
O8—C43	1.242 (4)	C47—H47C	0.9800
O7—C43	1.278 (5)	C48—H48A	0.9800
C43—C44	1.511 (5)	C48—H48B	0.9800
C11—C45	1.509 (5)	C48—H48C	0.9800
C10—C46	1.526 (5)	C5—H5	0.9500
C26—C47	1.514 (4)	C9—H9	0.9500
C27—C48	1.519 (5)	Cu1—N1	2.024 (3)
C4—C5	1.373 (5)	Cu1—N2	1.962 (2)
N1—C5	1.333 (4)	Cu1—O1	1.931 (2)
C1—C6	1.470 (4)	Cu1—O3	1.974 (2)
N3—C6	1.358 (4)	Cu1—O2	2.471 (2)
N2—C6	1.329 (4)	Cu1—O4	2.698 (3)
N2—C7	1.392 (4)	Cu2—N5	2.029 (2)
C7—C8	1.392 (4)	Cu2—N6	1.987 (3)
N3—C8	1.392 (4)	C100—O100	1.362 (9)
C8—C9	1.394 (4)	C200—O200	1.375 (9)
O100—H100	0.8400	C300—O300	1.388 (10)
C100—H101	0.9900	Cu2—O5	1.961 (2)
C100—H102	0.9900	Cu2—O7	1.955 (2)
C110—H111	0.9800	Cu2—O6	2.554 (3)
C110—H112	0.9800	Cu2—O8	2.546 (3)
C110—H113	0.9800		
			
C100—C110—H111	109.5	C17—C16—H16	120.6
C100—C110—H112	109.5	C15—C16—H16	120.6
H111—C110—H112	109.5	C16—C17—C18	118.4 (3)
C100—C110—H113	109.5	C16—C17—H17	120.8
H111—C110—H113	109.5	C18—C17—H17	120.8
H112—C110—H113	109.5	N4—C18—C17	123.7 (3)
O100—C100—C110	114.6 (9)	N4—C18—H18	118.2
O100—C100—H101	108.6	C17—C18—H18	118.2
C110—C100—H101	108.6	O4—C19—O3	122.5 (3)
O100—C100—H102	108.6	O4—C19—C20	120.3 (4)
C110—C100—H102	108.6	O3—C19—C20	117.2 (3)
H101—C100—H102	107.6	O4—C19—Cu1	72.71 (19)
C100—O100—H100	109.5	O3—C19—Cu1	49.90 (16)
C200—C210—H211	109.5	C20—C19—Cu1	166.3 (3)
C200—C210—H212	109.5	C19—C20—H20A	109.5
H211—C210—H212	109.5	C19—C20—H20B	109.5
C200—C210—H213	109.5	H20A—C20—H20B	109.5
H211—C210—H213	109.5	C19—C20—H20C	109.5
H212—C210—H213	109.5	H20A—C20—H20C	109.5
O200—C200—C210	113.2 (9)	H20B—C20—H20C	109.5
O200—C200—H201	108.9	O2—C21—O1	122.9 (3)
C210—C200—H201	108.9	O2—C21—C22	121.3 (4)
O200—C200—H202	108.9	O1—C21—C22	115.9 (3)
C210—C200—H202	108.9	C21—C22—H22A	109.5
H201—C200—H202	107.7	C21—C22—H22B	109.5
C200—O200—H200	109.5	H22A—C22—H22B	109.5
C300—C310—H311	109.5	C21—C22—H22C	109.5
C300—C310—H312	109.5	H22A—C22—H22C	109.5
H311—C310—H312	109.5	H22B—C22—H22C	109.5
C300—C310—H313	109.5	N6—C23—C24	108.5 (3)
H311—C310—H313	109.5	N6—C23—C28	131.3 (3)
H312—C310—H313	109.5	C24—C23—C28	120.2 (3)
O300—C300—C310	103.6 (9)	N7—C24—C25	131.3 (3)
O300—C300—H301	111.0	N7—C24—C23	106.5 (3)
C310—C300—H301	111.0	C25—C24—C23	122.2 (3)
O300—C300—H302	111.0	C26—C25—C24	117.5 (3)
C310—C300—H302	111.0	C26—C25—H25	121.2
H301—C300—H302	109.0	C24—C25—H25	121.2
C300—O300—H300	109.5	C25—C26—C27	121.0 (3)
O1—Cu1—N2	166.81 (11)	C25—C26—C47	118.9 (3)
O1—Cu1—O3	94.41 (9)	C27—C26—C47	120.1 (3)
N2—Cu1—O3	94.24 (10)	C28—C27—C26	120.5 (3)
O1—Cu1—N1	93.50 (10)	C28—C27—C48	119.3 (3)
N2—Cu1—N1	80.25 (10)	C26—C27—C48	120.1 (3)
O3—Cu1—N1	165.68 (10)	C27—C28—C23	118.6 (3)
O1—Cu1—C19	98.13 (10)	C27—C28—H28	120.7
N2—Cu1—C19	94.31 (10)	C23—C28—H28	120.7
O3—Cu1—C19	29.61 (10)	N6—C29—N7	111.9 (3)
N1—Cu1—C19	137.00 (11)	N6—C29—C30	118.5 (3)
O7—Cu2—O5	94.38 (10)	N7—C29—C30	129.5 (3)
O7—Cu2—N6	170.19 (10)	N5—C30—C31	121.2 (3)
O5—Cu2—N6	93.55 (10)	N5—C30—C29	111.1 (3)
O7—Cu2—N5	92.34 (10)	C31—C30—C29	127.7 (3)
O5—Cu2—N5	172.00 (11)	C32—C31—C30	119.3 (3)
N6—Cu2—N5	80.24 (10)	C32—C31—H31	120.3
C21—O1—Cu1	109.3 (2)	C30—C31—H31	120.3
C19—O3—Cu1	100.5 (2)	C33—C32—C31	119.3 (4)
C41—O5—Cu2	103.6 (2)	C33—C32—H32	120.4
C43—O7—Cu2	103.3 (2)	C31—C32—H32	120.4
C5—N1—C1	118.9 (3)	C32—C33—C34	119.0 (3)
C5—N1—Cu1	125.0 (2)	C32—C33—H33	120.5
C1—N1—Cu1	116.0 (2)	C34—C33—H33	120.5
C6—N2—C7	106.7 (2)	N5—C34—C33	122.4 (3)
C6—N2—Cu1	115.4 (2)	N5—C34—H34	118.8
C7—N2—Cu1	137.8 (2)	C33—C34—H34	118.8
C6—N3—C8	106.7 (3)	N7—C35—C36	114.1 (2)
C6—N3—C13	130.8 (3)	N7—C35—H35A	108.7
C8—N3—C13	121.9 (3)	C36—C35—H35A	108.7
C18—N4—C14	117.1 (3)	N7—C35—H35B	108.7
C34—N5—C30	118.9 (3)	C36—C35—H35B	108.7
C34—N5—Cu2	125.4 (2)	H35A—C35—H35B	107.6
C30—N5—Cu2	115.70 (19)	N8—C36—C37	122.2 (3)
C29—N6—C23	106.7 (3)	N8—C36—C35	114.7 (3)
C29—N6—Cu2	114.30 (19)	C37—C36—C35	123.0 (3)
C23—N6—Cu2	139.0 (2)	C36—C37—C38	119.8 (3)
C29—N7—C24	106.4 (2)	C36—C37—H37	120.1
C29—N7—C35	129.8 (3)	C38—C37—H37	120.1
C24—N7—C35	123.8 (3)	C39—C38—C37	118.5 (4)
C36—N8—C40	117.2 (3)	C39—C38—H38	120.8
N1—C1—C2	121.2 (3)	C37—C38—H38	120.8
N1—C1—C6	110.6 (3)	C38—C39—C40	118.7 (4)
C2—C1—C6	128.2 (3)	C38—C39—H39	120.7
C1—C2—C3	119.1 (3)	C40—C39—H39	120.7
C1—C2—H2	120.4	N8—C40—C39	123.6 (4)
C3—C2—H2	120.4	N8—C40—H40	118.2
C4—C3—C2	119.0 (3)	C39—C40—H40	118.2
C4—C3—H3	120.5	O6—C41—O5	122.7 (3)
C2—C3—H3	120.5	O6—C41—C42	120.7 (4)
C5—C4—C3	119.0 (3)	O5—C41—C42	116.6 (3)
C5—C4—H4	120.5	C41—C42—H42A	109.5
C3—C4—H4	120.5	C41—C42—H42B	109.5
N1—C5—C4	122.7 (3)	H42A—C42—H42B	109.5
N1—C5—H5	118.6	C41—C42—H42C	109.5
C4—C5—H5	118.6	H42A—C42—H42C	109.5
N2—C6—N3	111.7 (3)	H42B—C42—H42C	109.5
N2—C6—C1	117.7 (3)	O8—C43—O7	122.7 (3)
N3—C6—C1	130.6 (3)	O8—C43—C44	120.4 (4)
N2—C7—C8	108.1 (3)	O7—C43—C44	116.9 (3)
N2—C7—C12	131.4 (3)	C43—C44—H44A	109.5
C8—C7—C12	120.5 (3)	C43—C44—H44B	109.5
C7—C8—N3	106.7 (3)	H44A—C44—H44B	109.5
C7—C8—C9	121.9 (3)	C43—C44—H44C	109.5
N3—C8—C9	131.4 (3)	H44A—C44—H44C	109.5
C10—C9—C8	117.7 (3)	H44B—C44—H44C	109.5
C10—C9—H9	121.2	C11—C45—H45A	109.5
C8—C9—H9	121.2	C11—C45—H45B	109.5
C9—C10—C11	121.3 (3)	H45A—C45—H45B	109.5
C9—C10—C46	118.8 (4)	C11—C45—H45C	109.5
C11—C10—C46	119.9 (4)	H45A—C45—H45C	109.5
C12—C11—C10	120.3 (3)	H45B—C45—H45C	109.5
C12—C11—C45	119.2 (4)	C10—C46—H46A	109.5
C10—C11—C45	120.5 (3)	C10—C46—H46B	109.5
C11—C12—C7	118.3 (3)	H46A—C46—H46B	109.5
C11—C12—H12	120.9	C10—C46—H46C	109.5
C7—C12—H12	120.9	H46A—C46—H46C	109.5
N3—C13—C14	110.2 (3)	H46B—C46—H46C	109.5
N3—C13—H13A	109.6	C26—C47—H47A	109.5
C14—C13—H13A	109.6	C26—C47—H47B	109.5
N3—C13—H13B	109.6	H47A—C47—H47B	109.5
C14—C13—H13B	109.6	C26—C47—H47C	109.5
H13A—C13—H13B	108.1	H47A—C47—H47C	109.5
N4—C14—C15	123.0 (3)	H47B—C47—H47C	109.5
N4—C14—C13	116.2 (3)	C27—C48—H48A	109.5
C15—C14—C13	120.8 (3)	C27—C48—H48B	109.5
C16—C15—C14	118.9 (3)	H48A—C48—H48B	109.5
C16—C15—H15	120.6	C27—C48—H48C	109.5
C14—C15—H15	120.6	H48A—C48—H48C	109.5
C17—C16—C15	118.9 (3)	H48B—C48—H48C	109.5
			
C5—N1—C1—C2	0.6 (5)	C29—N6—C23—C24	0.8 (3)
Cu1—N1—C1—C2	177.3 (2)	Cu2—N6—C23—C24	−178.7 (2)
C5—N1—C1—C6	−179.0 (3)	C29—N6—C23—C28	−179.1 (3)
Cu1—N1—C1—C6	−2.3 (3)	Cu2—N6—C23—C28	1.4 (5)
N1—C1—C2—C3	0.6 (5)	C29—N7—C24—C25	177.8 (3)
C6—C1—C2—C3	−179.9 (3)	C35—N7—C24—C25	−4.4 (5)
C1—C2—C3—C4	−1.8 (5)	C29—N7—C24—C23	−1.0 (3)
C2—C3—C4—C5	2.0 (6)	C35—N7—C24—C23	176.8 (3)
C1—N1—C5—C4	−0.4 (5)	N6—C23—C24—N7	0.2 (3)
Cu1—N1—C5—C4	−176.9 (3)	C28—C23—C24—N7	−180.0 (3)
C3—C4—C5—N1	−0.8 (6)	N6—C23—C24—C25	−178.8 (3)
C7—N2—C6—N3	−1.6 (3)	C28—C23—C24—C25	1.1 (5)
Cu1—N2—C6—N3	−179.4 (2)	N7—C24—C25—C26	−177.8 (3)
C7—N2—C6—C1	178.4 (3)	C23—C24—C25—C26	0.8 (5)
Cu1—N2—C6—C1	0.5 (4)	C24—C25—C26—C27	−1.4 (5)
C8—N3—C6—N2	2.3 (3)	C24—C25—C26—C47	177.4 (3)
C13—N3—C6—N2	173.4 (3)	C25—C26—C27—C28	0.2 (5)
C8—N3—C6—C1	−177.6 (3)	C47—C26—C27—C28	−178.7 (3)
C13—N3—C6—C1	−6.6 (6)	C25—C26—C27—C48	180.0 (3)
N1—C1—C6—N2	1.2 (4)	C47—C26—C27—C48	1.1 (5)
C2—C1—C6—N2	−178.4 (3)	C26—C27—C28—C23	1.7 (5)
N1—C1—C6—N3	−178.9 (3)	C48—C27—C28—C23	−178.1 (3)
C2—C1—C6—N3	1.5 (6)	N6—C23—C28—C27	177.5 (3)
C6—N2—C7—C8	0.2 (3)	C24—C23—C28—C27	−2.3 (5)
Cu1—N2—C7—C8	177.3 (2)	C23—N6—C29—N7	−1.4 (3)
C6—N2—C7—C12	−178.2 (3)	Cu2—N6—C29—N7	178.20 (19)
Cu1—N2—C7—C12	−1.1 (6)	C23—N6—C29—C30	177.1 (3)
N2—C7—C8—N3	1.1 (3)	Cu2—N6—C29—C30	−3.3 (3)
C12—C7—C8—N3	179.7 (3)	C24—N7—C29—N6	1.5 (3)
N2—C7—C8—C9	−177.3 (3)	C35—N7—C29—N6	−176.1 (3)
C12—C7—C8—C9	1.4 (5)	C24—N7—C29—C30	−176.8 (3)
C6—N3—C8—C7	−2.0 (3)	C35—N7—C29—C30	5.6 (5)
C13—N3—C8—C7	−174.1 (3)	C34—N5—C30—C31	−1.7 (5)
C6—N3—C8—C9	176.1 (3)	Cu2—N5—C30—C31	175.8 (3)
C13—N3—C8—C9	4.1 (5)	C34—N5—C30—C29	179.5 (3)
C7—C8—C9—C10	−0.1 (5)	Cu2—N5—C30—C29	−3.0 (3)
N3—C8—C9—C10	−178.0 (3)	N6—C29—C30—N5	4.2 (4)
C8—C9—C10—C11	−0.7 (5)	N7—C29—C30—N5	−177.6 (3)
C8—C9—C10—C46	178.6 (3)	N6—C29—C30—C31	−174.5 (3)
C9—C10—C11—C12	0.3 (5)	N7—C29—C30—C31	3.7 (5)
C46—C10—C11—C12	−179.0 (3)	N5—C30—C31—C32	2.1 (5)
C9—C10—C11—C45	−178.6 (3)	C29—C30—C31—C32	−179.2 (3)
C46—C10—C11—C45	2.0 (5)	C30—C31—C32—C33	−0.9 (6)
C10—C11—C12—C7	0.9 (5)	C31—C32—C33—C34	−0.8 (7)
C45—C11—C12—C7	179.9 (3)	C30—N5—C34—C33	0.0 (5)
N2—C7—C12—C11	176.5 (3)	Cu2—N5—C34—C33	−177.2 (3)
C8—C7—C12—C11	−1.7 (5)	C32—C33—C34—N5	1.2 (6)
C6—N3—C13—C14	−86.0 (4)	C29—N7—C35—C36	−84.0 (4)
C8—N3—C13—C14	83.9 (4)	C24—N7—C35—C36	98.8 (3)
C18—N4—C14—C15	−0.9 (5)	C40—N8—C36—C37	−0.6 (5)
C18—N4—C14—C13	−179.6 (3)	C40—N8—C36—C35	175.8 (3)
N3—C13—C14—N4	50.3 (4)	N7—C35—C36—N8	144.9 (3)
N3—C13—C14—C15	−128.4 (3)	N7—C35—C36—C37	−38.7 (4)
N4—C14—C15—C16	1.0 (5)	N8—C36—C37—C38	−0.1 (6)
C13—C14—C15—C16	179.7 (3)	C35—C36—C37—C38	−176.3 (3)
C14—C15—C16—C17	−0.8 (5)	C36—C37—C38—C39	0.5 (6)
C15—C16—C17—C18	0.5 (5)	C37—C38—C39—C40	−0.1 (6)
C14—N4—C18—C17	0.6 (5)	C36—N8—C40—C39	1.1 (5)
C16—C17—C18—N4	−0.5 (5)	C38—C39—C40—N8	−0.7 (6)
Cu1—O3—C19—O4	−5.1 (3)	Cu2—O5—C41—O6	−6.3 (5)
Cu1—O3—C19—C20	174.3 (2)	Cu2—O5—C41—C42	173.3 (3)
Cu1—O1—C21—O2	6.6 (4)	Cu2—O7—C43—O8	−1.0 (4)
Cu1—O1—C21—C22	−173.4 (2)	Cu2—O7—C43—C44	177.3 (3)

### Bis(acetato-κO){5,6-dimethyl-2-(pyridin-2-yl)-1-[pyridin-2-yl)methyl]-1H-benzimidazole-κ2N2,N3}copper(II) ethanol hemisolvate (2) Hydrogen-bond geometry (Å, º)

**Table:**

*D*—H···*A*	*D*—H	H···*A*	*D*···*A*	*D*—H···*A*
O100—H100···O2^i^	0.84	2.25	3.010 (8)	152
O200—H200···O6	0.84	2.31	3.068 (14)	149
O300—H300···O6	0.84	2.41	3.13 (2)	144
C5—H5···O1	0.95	2.52	3.043 (4)	115
C5—H5···N8^ii^	0.95	2.52	3.279 (4)	137
C9—H9···O2^iii^	0.95	2.61	3.482 (5)	153
C12—H12···O3	0.95	2.51	3.171 (4)	127
C13—H13*A*···O2^iii^	0.99	2.53	3.497 (4)	167
C17—H17···O8^iv^	0.95	2.63	3.334 (4)	132
C20—H20*B*···O3^i^	0.98	2.61	3.576 (5)	169
C25—H25···O8^iv^	0.95	2.45	3.321 (4)	152
C28—H28···O5	0.95	2.50	3.188 (4)	129
C33—H33···O100^v^	0.95	2.51	3.202 (8)	130
C34—H34···O7	0.95	2.48	3.015 (4)	116
C35—H35*A*···O8^iv^	0.99	2.37	3.353 (4)	170
C42—H42*A*···O5^vi^	0.98	2.63	3.600 (6)	169

Symmetry codes: (i) −*x*+2, −*y*+1, −*z*+1; (ii) *x*+1, *y*, *z*; (iii) −*x*+2, −*y*, −*z*+1; (iv) −*x*+1, −*y*+1, −*z*; (v) *x*−1, *y*, *z*; (vi) −*x*+1, −*y*+2, −*z*.
